# Defective Cytochrome P450-Catalysed Drug Metabolism in Niemann-Pick Type C Disease

**DOI:** 10.1371/journal.pone.0152007

**Published:** 2016-03-28

**Authors:** Elena-Raluca Nicoli, Nada Al Eisa, Celine V. M. Cluzeau, Christopher A. Wassif, James Gray, Kathryn R. Burkert, David A. Smith, Lauren Morris, Stephanie M. Cologna, Cody J. Peer, Tristan M. Sissung, Constantin-Daniel Uscatu, William D. Figg, William J. Pavan, Charles H. Vite, Forbes D. Porter, Frances M. Platt

**Affiliations:** 1 Department of Pharmacology, University of Oxford, Oxford, United Kingdom; 2 Section of Molecular Dysmorphology, Eunice Kennedy Shriver National Institute of Child Health and Human Development, National Institutes of Health, Department of Health and Human Services, Bethesda, Maryland, United States of America; 3 Clinical Pharmacology Program, Medical Oncology Branch, National Cancer Institute, Department of Health and Human Services, Bethesda, Maryland, United States of America; 4 Department of Cellular and Molecular Biology, University of Medicine and Pharmacy of Craiova, Craiova, Romania; 5 Genetic Disease Research Branch, National Human Genome Research Institute, National Institutes of Health, Department of Health and Human Services, Bethesda, Maryland, United States of America; 6 School of Veterinary Medicine, University of Pennsylvania, Philadelphia, Pennsylvania, United States of America; The University of New South Wales, AUSTRALIA

## Abstract

Niemann-Pick type C (NPC) disease is a neurodegenerative lysosomal storage disease caused by mutations in either the *NPC1* or *NPC2* gene. NPC is characterised by storage of multiple lipids in the late endosomal/lysosomal compartment, resulting in cellular and organ system dysfunction. The underlying molecular mechanisms that lead to the range of clinical presentations in NPC are not fully understood. While evaluating potential small molecule therapies in *Npc1*^*-/-*^ mice, we observed a consistent pattern of toxicity associated with drugs metabolised by the cytochrome P450 system, suggesting a potential drug metabolism defect in NPC1 disease. Investigation of the P450 system in the context of NPC1 dysfunction revealed significant changes in the gene expression of many P450 associated genes across the full lifespan of *Npc1*^*-/-*^ mice, decreased activity of cytochrome P450 reductase, and a global decrease of multiple cytochrome P450 catalysed dealkylation reactions. *In vivo* drug metabolism studies using a prototypic P450 metabolised drug, midazolam, confirmed dysfunction in drug clearance in the *Npc1*^*-/-*^ mouse. Expression of the Phase II enzyme uridinediphosphate-glucuronosyltransferase (UGT) was also significantly reduced in *Npc1*^*-/-*^ mice. Interestingly, reduced activity within the P450 system was also observed in heterozygous *Npc1*^*+/-*^ mice. The reduced activity of P450 enzymes may be the result of bile acid deficiency/imbalance in *Npc1*^*-/-*^ mice, as bile acid treatment significantly rescued P450 enzyme activity in *Npc1*^*-/-*^ mice and has the potential to be an adjunctive therapy for NPC disease patients. The dysfunction in the cytochrome P450 system were recapitulated in the NPC1 feline model. Additionally, we present the first evidence that there are alterations in the P450 system in NPC1 patients.

## Introduction

Niemann-pick type C (NPC) disease is an autosomal recessive, neurodegenerative lysosomal disorder caused by mutation in either the *NPC1* or the *NPC2* gene [1]. A hallmark of NPC at the cellular level is the accumulation of a wide range of lipids including unesterified cholesterol, glycosphingolipids, sphingomyelin and sphingosine in late endosomes/lysosomes (LE/Lys) [1, 2]. This storage of lipids in LE/Lys leads to a deficiency of lipids trafficking into key biosynthetic pathways, ultimately resulting in a myriad of cellular dysfunction [1, 2].

NPC1 disease clinically is heterogeneous in terms of the age of onset and clinical presentation, typically presenting in infancy/childhood. Symptoms include hepatosplenomegaly, vertical supranuclear ophthalmoplegia, ataxia and dementia leading to premature death. Adult-onset forms of NPC also occur and typically present with psychiatric symptoms, dementia and ataxia [1, 3]. Recent studies suggest the prevalence of the late-onset forms of NPC1 are likely greater than previously recognized [4]. Currently, the only approved therapy for NPC1 disease is miglustat, a small molecule inhibitor of glycosphingolipid biosynthesis. Miglustat is approved in Europe and multiple other regions for treating the neurological manifestations of NPC1 disease but is currently not approved by the FDA [5–8].

The *Npc1*^*-/-*^ (BALB/cNctr-*Npc1m1N*/J) mouse recapitulates many of the features of the human disease: mutants are phenotypically normal at birth, although gene expression profiling by microarray has detected significant changes as early as one week of age [9]. By 5–6 weeks of age *Npc1*^*-/-*^ mice present with progressive neurological symptoms including tremor and ataxia and reach end stage disease by 12 weeks of age [10]. Multiple treatments have shown varying degrees of efficacy in the *Npc1*^*-/-*^ mouse including pharmacological compounds such as ibuprofen, calcium modulating agents including curcumin, c-abl tyrosine kinase inhibitors imatinib mesylate, and synthetic agonists targeting nuclear receptors, such as liver X receptor (LXR) and pregnane X receptor (PXR) [11–15]. Studies have also shown a significant therapeutic benefit of 2-hydroxypropyl-β-cyclodextrin (HPBCD) in the reduction of lipid storage, thereby ameliorating neurodegeneration and extending the life span of both the murine and feline models of NPC1 disease. HPBCD is currently in a phase I clinical study (NCT01747135) at the National Institutes of Health (NIH) [16].

The liver in NPC1 disease has a considerable lipid storage burden, including elevated levels of free cholesterol, with liver pathology relatively common in patients, manifesting in the neonatal period as cholestatic jaundice [1, 17]. This typically resolves by the age of 3 months and can be difficult to differentiate from physiological jaundice present in many new-borns. However, in approximately 10% of NPC patients this neonatal liver presentation progresses to liver failure leading to premature death [17–19]. Clinical management of cholestatic jaundice as well as primary biliary cirrhosis includes bile acid therapy, specifically the use of ursodeoxycholic acid (UDCA) [20]. Bile acid therapy has been effective in some cases of NPC1 (F.D.Porter) [21–23] and is discontinued once the hepatic phase of the disease has resolved.

During screening of potentially therapeutic small molecules in *Npc1*^-/-^ mice, we consistently observed increased toxicity with drugs that undergo hepatic metabolism, suggesting a potential impairment of the cytochrome P450 system [24–27]. The P450 system is regulated in a multifaceted manner in part by oxysterols and bile acids [28–30]. The sequestration of cholesterol in the late endosome/lysosome of NPC1 patients results in altered regulation and synthesis of both oxysterols and bile acids [31–34] thereby potentially impairing P450 gene regulation [29]. In this study, we have therefore investigated drug metabolism in detail and found that expression and function of the P450 system is significantly impaired in the murine and feline models of NPC1 disease, and provide the first evidence that this system is potentially altered in NPC1 patients. This dysfunction is likely caused by a bile acid imbalance, supported by the finding that P450 enzyme activity can be restored with bile acid supplementation using UDCA.

## Results

Our laboratory has tested numerous small molecule therapies as possible therapeutic interventions in *Npc1*^*-/-*^ mice [12]. During these trials it was observed that compounds that are primarily subject to first-pass metabolism in the liver were associated with reduced tolerability and toxicity [12] (Table 1). When lower doses were used positive therapeutic benefits were observed [12] (Table 1). The reduced doses required for ibuprofen, vinpocetine and curcumin in *Npc1*^*-/-*^ mice are summarised along with the normal dose used in other mouse strains (Table 1). In contrast, drugs such as miglustat, which are renally excreted intact, could be used at doses comparable to those reported in other murine models and strains [12, 35].

**Table 1:** Examples of therapeutic drugs requiring lower dosing in *Npc1*^-/-^ mice.

Drug	Normally Effective Dose (mg/kg/day)	Adjusted Reduced Dose (mg/kg/day) in *Npc1*^*-/-*^ mice	Notes	References
Curcumin	300–1250	150	Liver metabolized; Inhibits some CYP450 isoforms.	[24–26]
Ibuprofen	300–800	100	Cytochrome P450 metabolized.	[27]
Vinpocetine	10	2	Cytochrome P450 metabolized.	[73]

Examples of therapeutic agents in *Npc1*^-/-^ mice, with their optimal doses reported in other mouse models and the reduced doses required in *Npc1*^*-/-*^ mice to avoid toxicity.

**Fig 1:**
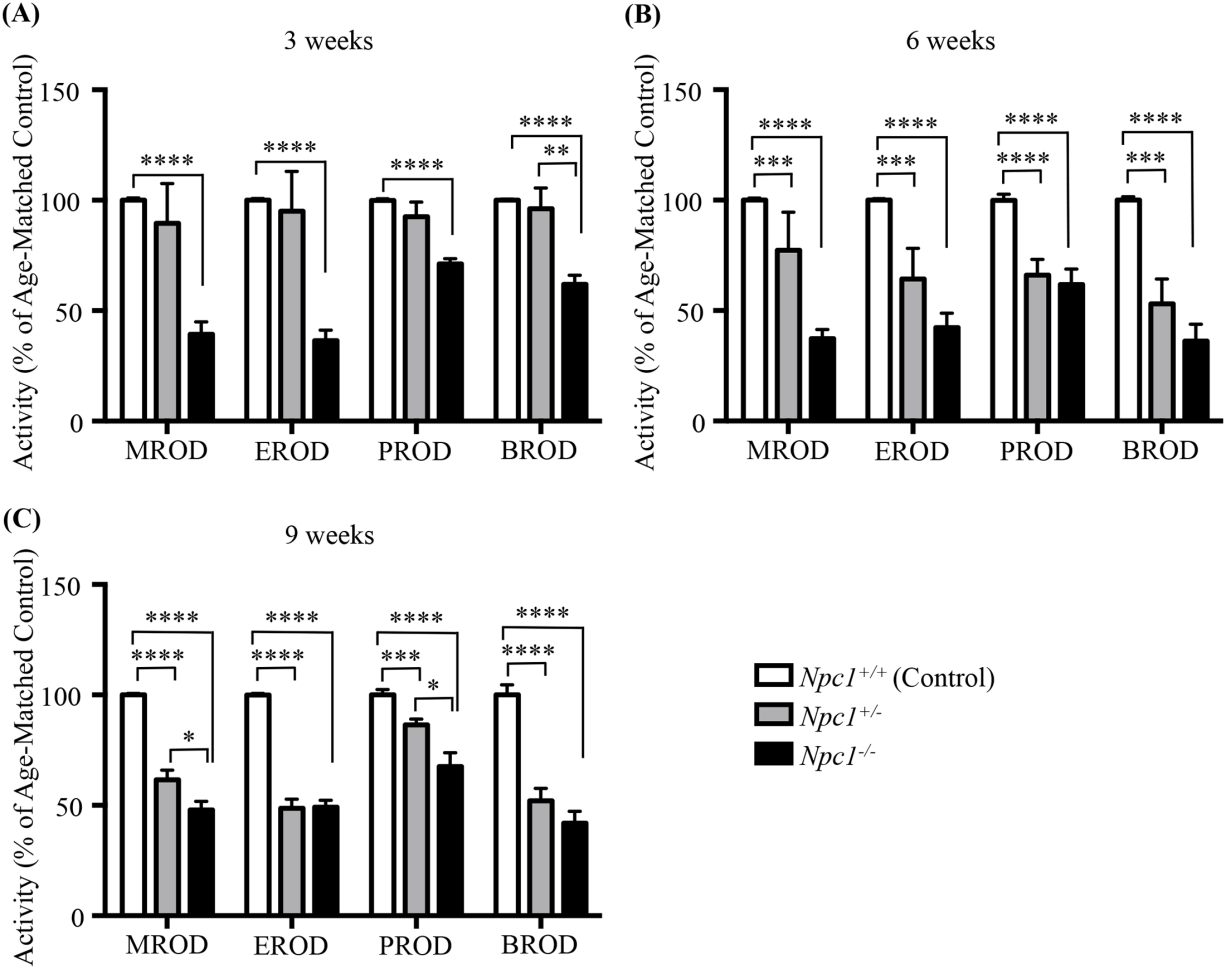
P450 enzymatic O-dealkylation activity assayed in *Npc1*^*+/+*^, *Npc1*^*+/-*^, and *Npc1*^*-/-*^ mice at 3,6, and 9 weeks of age. P450 O-dealkylation enzymatic activity of: MROD, methoxyresorufin-O-dealkylation; EROD, ethoxyresorufin-O-dealkylation; PROD, pentoxyresorufin-O-dealkylation, and BROD, benzoxyresorufin-O-dealkylation in *Npc1*^*+/+*^ (white), *Npc1*^*+/-*^ (grey), and *Npc1*^*-/-*^ (black) mice. Activity shown as percent of age-matched controls at 3 weeks (**A**), 6 weeks (**B**) and 9 weeks (**C**) of age. Data are presented as mean ± SEM, n = 6 (3 males and 3 females), * *p—*value < 0.05, ** *p—*value < 0.01, *** *p—*value < 0.001, **** *p—*value < 0.0001, calculated using two-tailed unpaired nonparametric Mann-Whitney test.

To test the hypothesis that the increased drug toxicity observed in *Npc1*^*-/-*^ mice is the result of perturbations in the cytochrome P450 system, we investigated the enzymatic activities of the methoxyresorufin-O-dealkylation (MROD), ethoxyresorufin-O-dealkylation (EROD), pentoxyresorufin-O-dealkylation (PROD) and, benzoxyresorufin-O-dealkylation (BROD) in *Npc1* mutant, heterozygous and control littermates mice at 3, 6, and 9 weeks of age. Significant reductions in all four enzymatic reactions were noted in *Npc1*^*-/-*^ mice at all time points tested (*p* < 0.0001) (Fig 1A–1C and *p* values and enzymatic activities are provided in Table 2). Heterozygous *Npc1* mice demonstrated no statistical differences in any of the four-enzymatic activities compared with control littermates at 3 weeks of age (Fig 1A and Table 2). However, *Npc1*^*+/-*^ mice had significant reductions in MROD, EROD, PROD and BROD by 6 weeks of age (Fig 1B and Table 2), and remained significantly reduced at 9 weeks of age (Fig 1C and Table 2). In agreement with these findings, cytochrome C reductase activity was significantly reduced at 9 weeks of age in the *Npc1*^*-/-*^ and *Npc1*^*+/-*^ mice compared to control littermate mice ((4.9 ± 0.2 vs. 11.6 ± 1.2 pmol/min/mg *p* = 0.032) (4.0 ± 0.3 vs. 11.6 ± 1.2 pmol/min/mg *p* = 0.008) respectively) (S1 Fig). No differences were noted between the sexes of any genotype at any time point.

**Table 2:** Enzyme activity rates, and relevant statistics of the P450 activity assays.

P450 activity rates	3 weeks				6 weeks				9 weeks			
	*Npc1*^*+/-*^		*Npc1*^*-/-*^		*Npc1*^*+/-*^		*Npc1*^*-/-*^		*Npc1*^*+/-*^		*Npc1*^*-/-*^	
	% of Age-Matched Control ± SEM	*p*—value	% of Age-Matched Control ± SEM	*p*—value	% of Age-Matched Control ± SEM	*p*—value	% of Age-Matched Control ± SEM	*p*—value	% of Age-Matched Control ± SEM	*p*—value	% of Age-Matched Control ± SEM	*p*—value
**MROD**	89.5 ± 18.0	0.0901	37.3 ± 4.2	< 0.0001	77.3 ± 17.3	0.0005	37.3 ± 4.2	< 0.0001	61.4 ± 4.5	< 0.0001	47.9 ± 3.9	< 0.0001
**EROD**	95.0 ± 18.0	0.9291	42.3 ± 6.4	< 0.0001	64.3 ± 13.9	0.0004	42.3 ± 6.4	< 0.0001	48.5 ± 4.1	< 0.0001	49.2 ± 3.1	< 0.0001
**PROD**	92.5 ± 6.6	> 0.9999	61.8 ± 7.0	< 0.0001	66.1 ± 7.1	< 0.0001	61.8 ± 7.0	< 0.0001	86.3 ± 2.6	0.0001	67.6 ± 6.2	< 0.0001
**BROD**	96.2 ± 9.3	> 0.9999	36.1 ± 7.6	< 0.0001	53.1 ± 11.2	0.0004	36.1 ± 7.6	< 0.0001	51.9 ± 5.7	< 0.0001	41.9 ± 5.4	< 0.0001

Enzyme activity rates of MROD, EROD, PROD and BROD at 3, 6 and 9 weeks of age in *Npc1*^*+/-*^ and *Npc1*^*-/-*^ mice as compared with wild type age-matched control. Data are presented as mean ± SEM, n = 6, calculated using two-tailed unpaired nonparametric Mann-Whitney test. All data was standardized to *Npc1*^*+/+*^ mouse data with enzyme activity rates of 100%.

**Fig 2:**
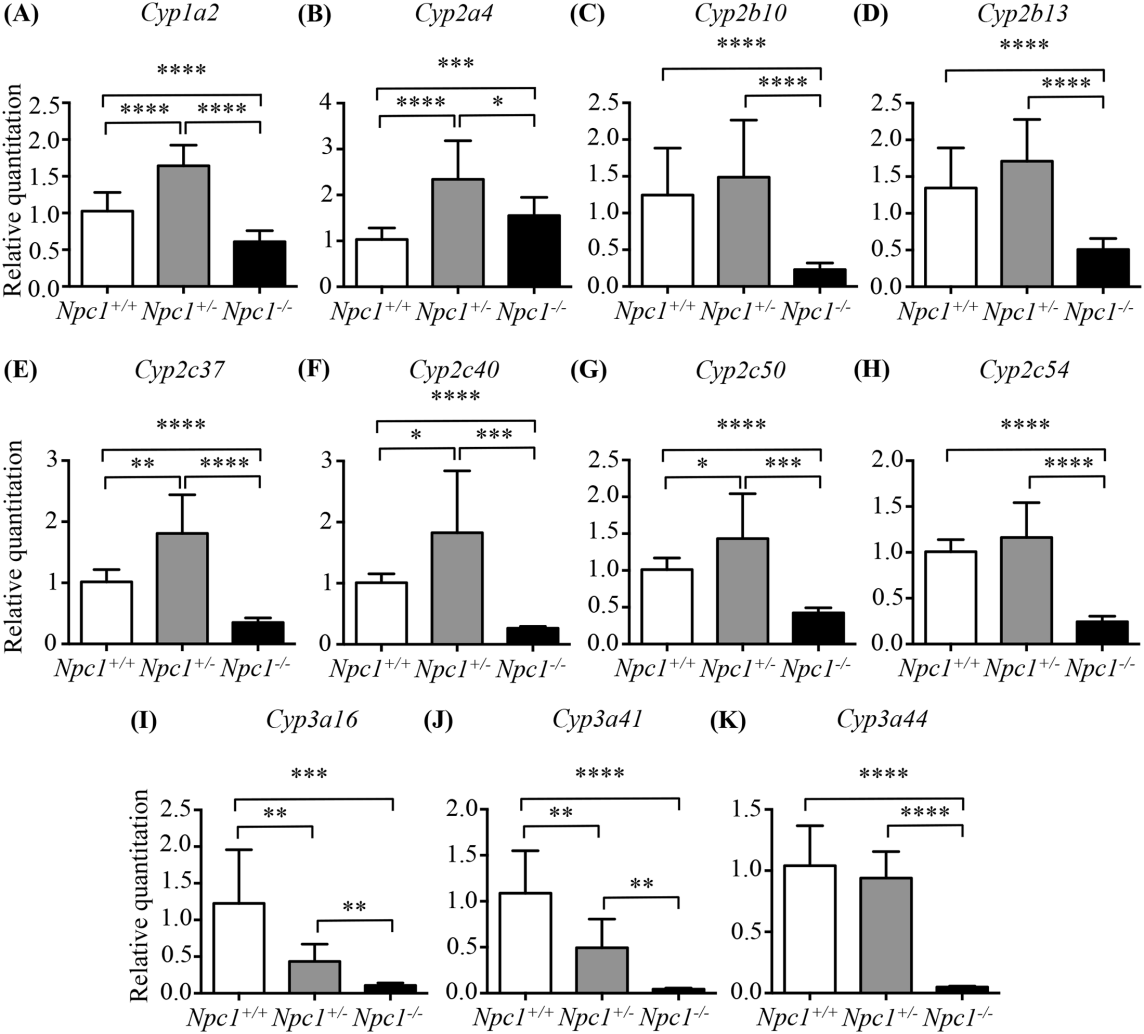
Validation of 11 CYP genes expression by qPCR in *Npc1*^*+/+*^, *Npc1*^*+/-*^, *and Npc1*^*-/-*^ mice at 9 weeks of age. Validation was performed in females to recapitulate the microarray studies. White columns for *Npc1*^*+/+*^ samples, grey columns for *Npc1*^*+/-*^, and black columns for *Npc1*^*-/-*^ mice. Data are presented as mean ± SD, n = 4–6. * *p—*value < 0.05, ** *p—*value < 0.01, *** *p—*value < 0.001, **** *p—*value < 0.0001, calculated using two-tailed unpaired t test, with Welch’s correction as necessary.

Previously, microarray analysis using mRNA isolated from female livers of *Npc1*^*-/-*^ and control littermate mice over the mutant mouse's life span revealed 62 of 101 known genes of the cytochrome P450 family as having modified expression [9] (S1 Table). In total, 44 of these 62 genes were down regulated at a minimum of one time point within the life span of the *Npc1*^*-/-*^ mouse, while 14 of the 44 were significantly down regulated at all time points. Genes belonging to subfamilies 1 to 3 (42 genes in all), the products of which are primarily responsible for drug metabolism, were particularly affected, with the vast majority being down-regulated. We therefore performed qPCR on mRNA isolated from 9-week-old female livers to validate the changes in the expression array data for 11 of these genes (Fig 2): *Cyp1a2*, *Cyp2a4*, *Cyp2b10*, *Cyp2b13*, *Cyp2c37*, *Cyp2c40*, *Cyp2c50*, *Cyp2c54*, *Cyp3a16*, *Cyp3a41* and *Cyp3a44*. A subset of 9 genes was assayed across all time points and compared directly with the microarray data (S2 Fig). All assayed genes showed high concordance with the microarray data. The expression of most mouse isozymes of the Cyp1a, Cyp2b and Cyp3a subfamilies were down regulated in *Npc1*^*-/-*^ compared to *Npc1*^*+/+*^ animals to differing degrees (Fig 2 and S1 Table). The Cyp2a subfamily was found down regulated at early time points in the microarray data (S1 Table). *Cyp2a4* showed a modest increase in expression in the 9-week-old *Npc1*^*-/-*^ compared to controls (Fig 2). Out of the 11 isozymes tested in heterozygous animals only the expression of *Cyp3a16* and *Cyp3a41* were reduced at 9 weeks compared to control mice (Fig 2). Nine-week-old *Npc1*^*+/-*^ mice presented a variable pattern of expression with 5 genes having increase expression (*Cyp1a2*, *Cyp2a4*, *Cyp2c37*, *Cyp2c40*, and *Cyp2c50*), 4 unchanged (*Cyp2b10*, *Cyp2b13*, *Cyp2c54*, and *Cyp3a44*), and 2 decrease (*Cyp3a16*, and *Cyp3a41*) all comparisons in relation to control female littermates. This data may point toward a functionally insufficient increase in some Cyp gene family members in the heterogynous animal as evident by the decreased activities (Fig 1C).

**Fig 3:**
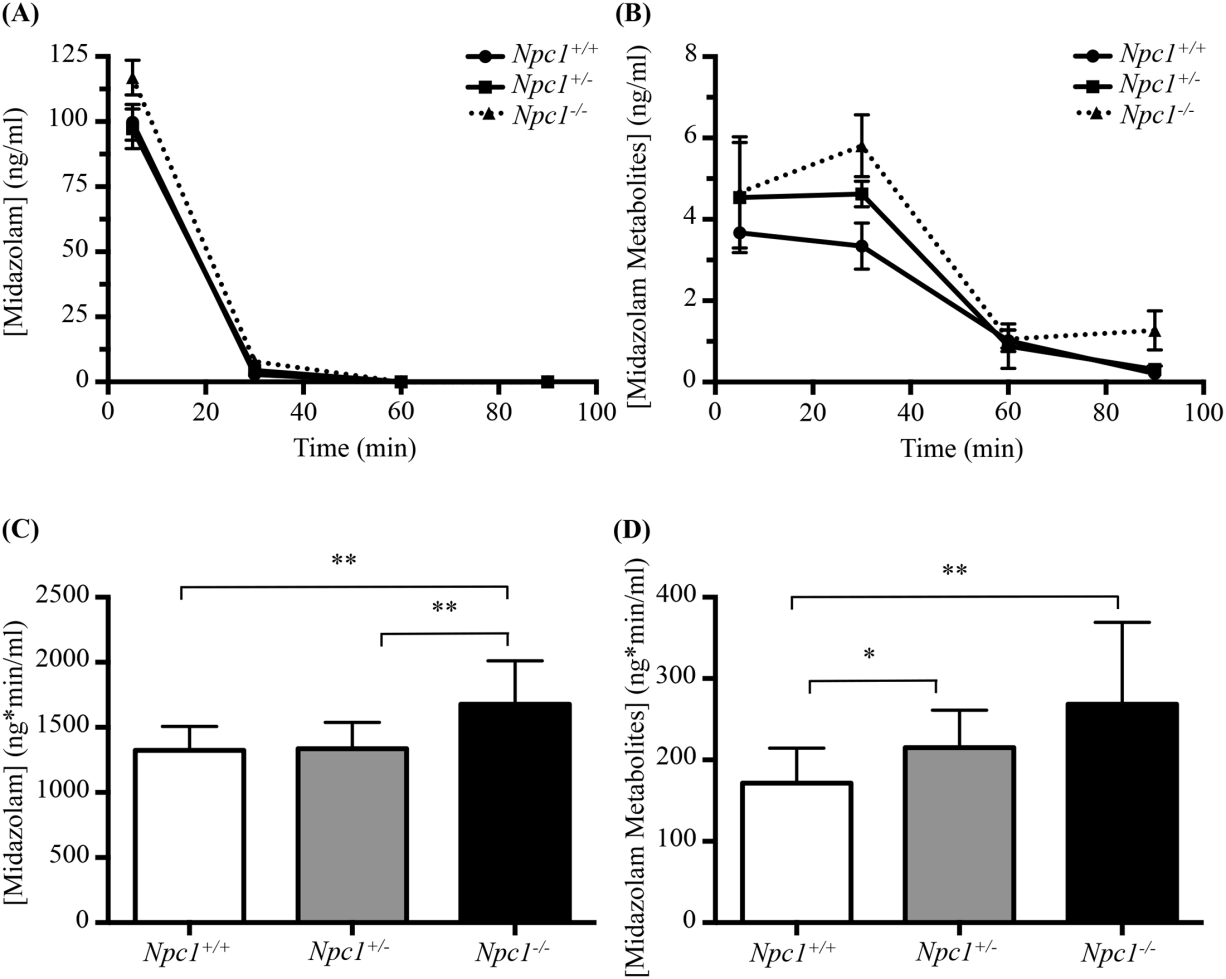
*In vivo* assay of Midazolam metabolism *Npc1*^*+/+*^, *Npc1*^*+/-*^, *and Npc1*^*-/-*^ mice. (**A**) Midazolam plasma concentration (in ng/ml) as a function of time. (**B**) Midazolam metabolites plasma concentration (in ng/ml) as a function of time. (**C**) and (**D**) Differences in exposure between genotypes. Data are presented as mean ± SD for each genotype, n = 3. * *p—*value <0.05, ** *p—*value < 0.01, calculated using two-tailed, unpaired t test with Welch’s correction as necessary and one-way ANOVA.

To determine whether there was a functional defect in drug metabolism, we evaluated the *in vivo* rate of midazolam metabolism. Midazolam was selected for this purpose due to its well-established pharmacokinetics, easily identifiable metabolites and rapid distribution/clearance [36–39]. Midazolam itself was cleared rapidly; with plasma concentration falling to below 1 ng/ml (limit of detection) 30 minutes post IV administration (Fig 3A). Thirty minutes after administration elevated plasma midazolam metabolite levels were observed in *Npc1*^-/-^ mice relative to control mice, with an intermediate increase observed in *Npc1*^*+/-*^ animals (Fig 3B).

There were significant differences in midazolam exposure (area under the plasma concentration vs time curves, calculated using Bailer’s Method for destructive sampling) between *Npc1*^*+/+*^ (1324.7 ± 183.4 ng*min/ml) and *Npc1*^*-/-*^ (1677.9 ± 333.4 ng*min/ml; *p* = 0.005), and significant differences between *Npc1*^+/-^ (1336.0 ± 201.7 ng*min/ml) and *Npc1*^*-/-*^ mice (*p* = 0.007) (Fig 3C). There was no difference (*p* = 0.89) in midazolam exposure between *Npc1*^+/+^ and *Npc1*^*+/-*^. For the metabolite exposures, there were statistically significant differences between *Npc1*^*+/+*^ (171.6 ± 42.9 ng*min/ml) and *Npc1*^*+/-*^ (215.1 ± 46.1 ng*min/ml; *p* = 0.030), as well as between *Npc1*^*+/+*^ and *Npc1*^*-/-*^ (268.6 ± 100.7 ng*min/ml; *p* = 0.008) (Fig 3D). There was no statistically significant difference in metabolite levels between *Npc1*^*+/-*^ and *Npc1*^*-/-*^ mice (*p* = 0.12).

Additionally, multiple UGT isoforms have significantly lower expression levels in *Npc1*^*-/-*^ compared to *Npc1*^*+/+*^ (S2 Table). This finding might explain the differences we observed in the rate of clearance of midazolam metabolites *in vivo* in *Npc1*^*-/-*^ mice, as UGT enzymes further metabolize CYP3A4-mediated hydroxymidazolam metabolites via Phase II O-linked or N-linked glucuronidation by UGT1A4, 2B4 and 2B7 [40].

**Fig 4:**
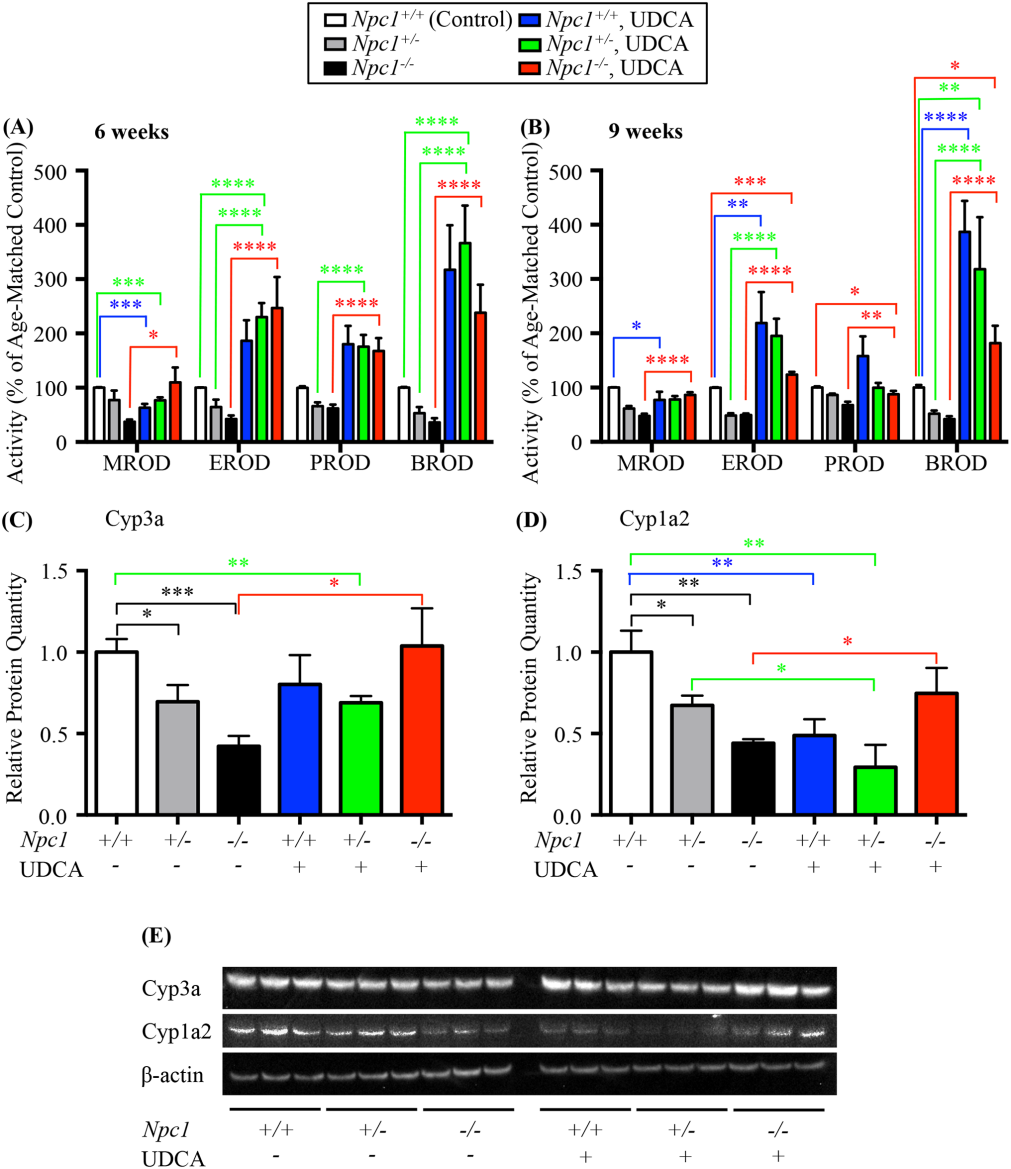
Effect of UDCA bile acid supplementation. (**A**) and (**B**) effects of UDCA bile acid supplementation on the activity of CYP450 catalysed reactions in the *Npc1* mouse model of plus or minus 0.5% ursodeoxycholic acid (UDCA, w/w) measured at (**A**) 6 weeks and (**B**) 9 weeks of age, shown as a function of percent of age-matched control littermates. Methoxyresorufin-O-dealkylation (MROD); ethoxyresorufin-O-dealkylation (EROD); pentoxyresorufin-O-dealkylation (PROD); benzoxyresorufin-O-dealkylation (BROD). Data are presented as mean ± SEM, n = 6 (3 males and 3 females), * *p—*value < 0.05, ** *p—*value < 0.01, *** *p—*value < 0.001, **** *p—*value < 0.0001 calculated using an unpaired nonparametric test Mann-Whitney test. (**C**) Relative protein quantitation of Cyp3a isolated from liver of 6-week old male mice found a significant decrease in *Npc1*^*+/-*^ and *Npc1*^*-/-*^ untreated mice compared *Npc1*^*+/+*^ untreated mice. UDCA treatment restored *Npc1*^*-/-*^ Cyp3a levels to that of *Npc1*^*+/+*^ mice while have no effect on the *Npc1*^*+/-*^ or *Npc1*^*+/+*^ Cyp3a values. (**D**) Relative protein quantitation of Cyp1a2 isolated from liver of 6-week old male mice found a significant decrease in *Npc1*^*+/-*^ and *Npc1*^*-/-*^ untreated mice compared *Npc1*^*+/+*^ untreated mice. UDCA treatment restored *Npc1*^*-/-*^ Cyp1a2 levels to that of *Npc1*^*+/+*^ mice while decreasing the levels for *Npc1*^*+/-*^ and *Npc1*^*+/+*^ Cyp1a2 values. Data are presented as mean ± SD, n = 3. * *p—*value < 0.05, ** *p—*value < 0.01, *** *p—*value < 0.001 calculated using two-tailed, unpaired t test. (**F**) Representative western blot analysis of Cyp3a and Cyp1a2 in the mouse model of *Npc1* plus or minus UDCA treatment.

One potential mechanism explaining the reduced expression of P450-related genes is a bile acid deficiency or imbalance, as bile acid levels are part of the multifactorial regulation of the P450 system [41]. We therefore treated *Npc1*^*-/-*^, *Npc1*^*+/-*^ and control littermate (*Npc1*^*+/+*^) mice of both genders with UDCA (0.5% of diet) from three weeks of age and measured P450 enzyme activity at 6 and 9 weeks of age. UDCA therapy resulted in the complete normalization or overexpression of P450 enzyme activities (Fig 4A and 4B and Tables 3 and 4). Interestingly, this normalization was consistently greater at 6 weeks of age, while the absolute percentage increase was reduced at 9 weeks compared to the 6-week values. This is not an unexpected result as the disease is continuing to progress over time leading to a fibrotic liver [42]. As such these findings likely implicates reduced bile acid levels or imbalances in bile acid species in NPC1 as an important mechanism underlying the drug metabolism defect.

**Table 3:** Enzyme activity rates, and relevant statistics of the P450 activity assays after treatment with UDCA as compared to untreated in *Npc1*^*+/+*^ mice.

P450 activity rates	6 weeks						9 weeks					
	*Npc1*^*+/+*^, UDCA		*Npc1*^*+/-*^, UDCA		*Npc1*^*-/-*^, UDCA		*Npc1*^*+/+*^, UDCA		*Npc1*^*+/-*^, UDCA		*Npc1*^*-/-*^, UDCA	
	% of Age-Matched Control ± SEM	*p*—value	% of Age-Matched Control ± SEM	*p*—value	% of Age-Matched Control ± SEM	*p*—value	% of Age-Matched Control ± SEM	*p*—value	% of Age-Matched Control ± SEM	*p*—value	% of Age-Matched Control ± SEM	*p*—value
**MROD**	63.2 ± 7.0	0.0004	76.8 ± 5.4	0.0005	109.8 ± 27.5	0.0892	77.5 ± 14.7	0.0204	78.2 ± 6.4	0.2406	86.6 ± 4.9	0.2273
**EROD**	186.4 ± 37.9	0.0964	230.4 ± 25.7	< 0.0001	246.7 ± 57.2	0.0901	218.9 ± 57.3	0.0037	195.3 ± 31.8	0.901	124.0 ± 5.0	0.0004
**PROD**	180.2 ± 33.7	0.1201	175.8 ± 21.7	0.0785	167.7 ± 23.9	0.3517	158.2 ± 36.3	0.3682	99.8 ± 9.0	0.8489	87.7 ± 6.1	0.0105
**BROD**	317.5 ± 81.9	0.1416	366.6 ± 69.0	< 0.0001	238.0 ± 51.7	0.0901	387.0 ± 56.7	< 0.0001	318.0 ± 95.9	0.0035	181.9 ± 32.3	0.0288

Enzyme activity rates of MROD, EROD, PROD and BROD at 6 and 9 weeks of age in *Npc1*^*+/+*^, *Npc1*^*+/-*^ and *Npc1*^*-/-*^ mice treated with 0.5% UDCA as compared with untreated wild type age-matched control. Data are presented as mean ± SEM, n = 6, calculated using two-tailed unpaired nonparametric Mann-Whitney test. All data was standardized to *Npc1*^*+/+*^ mouse data with enzyme activity rates of 100%.

**Table 4:** Enzyme activity rates, and relevant statistics of the P450 activity assays after treatment with UDCA intergenotypic comparisons.

P450 activity rates	6 weeks				9 weeks			
	*Npc1*^*+/-*^, *UDCA vs Npc1*^*+/-*^		*Npc1*^*-/-*^, *UDCA vs Npc1*^*-/-*^		*Npc1*^*+/-*^, *UDCA vs Npc1*^*+/-*^		*Npc1*^*-/-*^, *UDCA vs Npc1*^*-/-*^	
	% Change	*p*—value	% Change	*p*—value	% Change	*p*—value	% Change	*p*—value
**MROD**	-0.4	0.0901	72.5	0.0363	16.8	0.0856	38.7	< 0.0001
**EROD**	166.1	< 0.0001	204.3	< 0.0001	146.7	< 0.0001	74.8	< 0.0001
**PROD**	109.7	< 0.0001	105.9	< 0.0001	13.5	0.3269	20.1	0.0068
**BROD**	313.5	< 0.0001	201.9	< 0.0001	266.1	< 0.0001	139.9	< 0.0001

Percent change of enzyme activity rates for MROD, EROD, PROD and BROD at 6 and 9 weeks of age in *Npc1*^*+/-*^ and *Npc1*^*-/-*^ mice treated with 0.5% UDCA as compared with *Npc1*^*+/-*^ and *Npc1*^*-/-*^ mice respectively. Data are presented as mean ± SEM, n = 6, calculated using two-tailed unpaired nonparametric Mann-Whitney test.

*Npc1*^-/-^ mice treated with UDCA had a moderate increase in MROD activity (expressed as percentage of age-matched controls) at 6 weeks of age compared with untreated *Npc1*^-/-^ mice. MROD activity is not increased significantly in heterozygous *Npc1*^+/-^ mice treated with UDCA, at either 6 or 9 weeks of age relative to untreated *Npc1*^+/-^ mice. Both *Npc1*^+/+^ and *Npc1*^*+/-*^ mice treated with UDCA have reduced MROD activity at 6 weeks of age compared with untreated *Npc1*^*+/+*^(control) mice, while no reduction was noted by 9 weeks in the *Npc1*^*+/-*^ mice treated with UDCA (Fig 4A and 4B and Tables 3 and 4).

EROD, PROD, and BROD enzymatic activities were all significantly increased at 6 weeks in *Npc1*^*-/-*^ mice treated with UDCA, but this effect was diminished at 9 weeks when compared with the untreated *Npc1*^*-/-*^ mice. The same trend was apparent in the *Npc1*^*+/-*^ treated mice where at 6 weeks EROD, PROD, and BROD were significantly increased (Fig 4A and 4B and Tables 3 and 4). However, in late stage disease (9 weeks of age) no significant increase in PROD activity was detected, with a moderate increase in both EROD and BROD enzymatic activity in the *Npc1*^*+/-*^ mice when compared to untreated *Npc1*^*+/-*^ mice, similar to what was observed at 9 weeks in the treated *Npc1*^*-/-*^ mice.

The effects of UDCA treatment resulted in the increased protein expression levels of two critical P450 family members, Cyp3a and Cyp1a2 as depicted in the western blot (Fig 4E) and represented in the relative protein quantitation (Fig 4C and 4D). Treatment with UDCA restored Cyp3a protein levels in *Npc1*^-/-^ mice to *Npc1*^+/+^ control levels (*Npc1*^-/-^ UDCA treated 1.04 ± 0.13 vs *Npc1*^+/+^ untreated 1.00 ± 0.04, ns; *Npc1*^-/-^ UDCA treated 1.04 ± 0.13 vs *Npc1*^-/-^ untreated 0.42 ± 0.03, *p* = 0.011), while having no effects on Cyp3a protein levels in *Npc1*^+/+^ or *Npc1*^*+/-*^ mice (Fig 4E and 4C). In a similar fashion UDCA treatment resulted in increased Cyp1a2 levels in the *Npc1*^-/-^ mice (*Npc1*^-/-^ UDCA treated 0.75 ± 0.09 vs *Npc1*^-/-^ untreated 0.44 ± 0.01, *p* = 0.029), however a significant decrease of Cyp1a2 levels was noted in UDCA *Npc1*^+/+^ and *Npc1*^*+/-*^ treated mice (Fig 4E and 4D) compared with the untreated *Npc1*^+/+^ or *Npc1*^*+/-*^ mice (*Npc1*^+/+^ UDCA treated 0.49 ± 0.06 vs *Npc1*^+/+^ untreated 1.00 ± 0.08, *p* = 0.006; *Npc1*^+/-^ UDCA treated 0.29 ± 0.08 vs *Npc1*^+/+^ untreated 1.00 ± 0.08, *p* = 0.003; *Npc1*^+/-^ UDCA treated 0.29 ± 0.08 vs *Npc1*^+/-^ untreated 0.67 ± 0.03, *p* = 0.012).

### Effects of UDCA on body weight, rearing and tremor of *Npc1*^*-/-*^ mice

**Fig 5:**
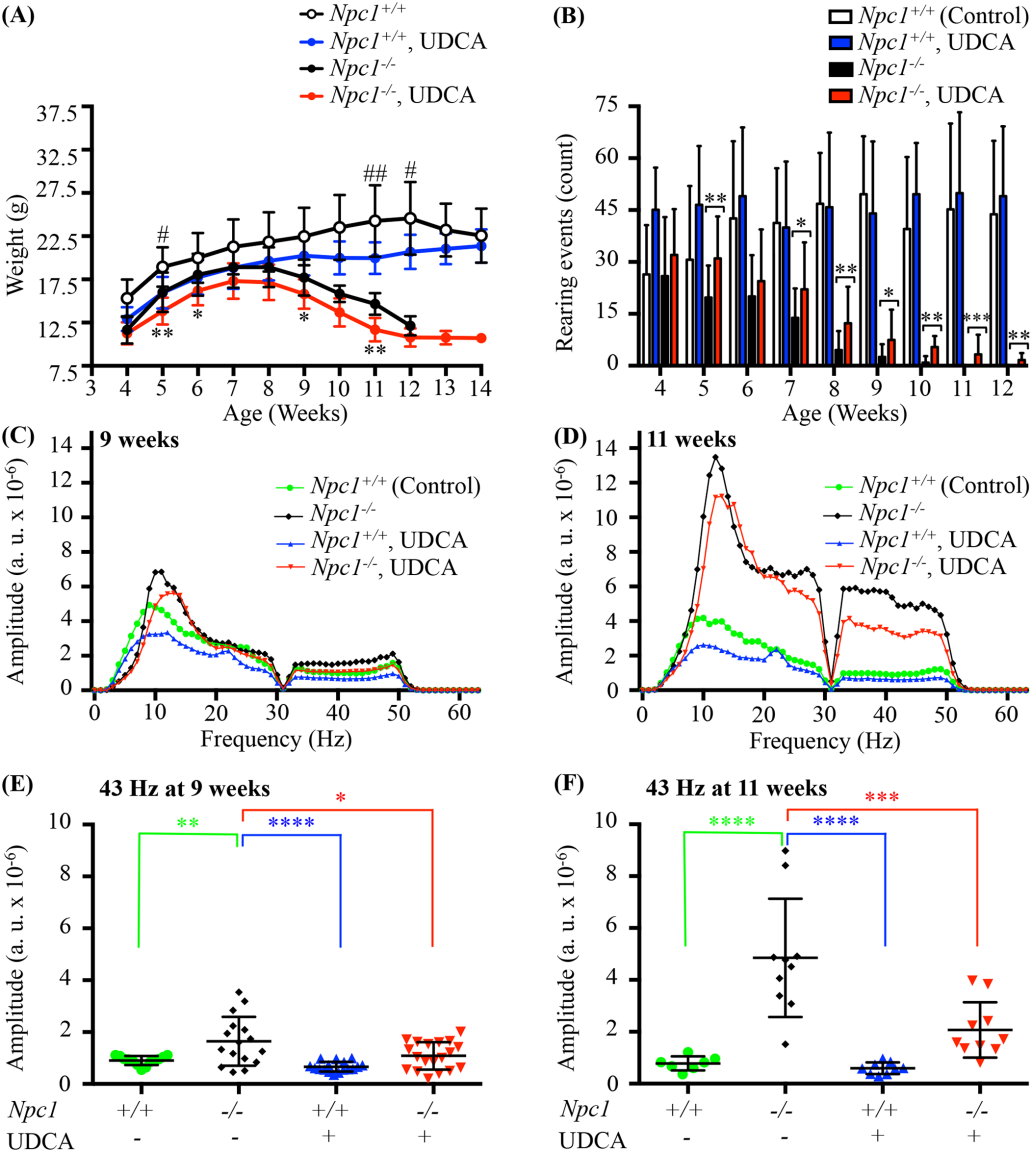
Effect of UDCA on body weight, rearing and tremor of *Npc1* mice. (**A**) Effects of UDCA on weight of *Npc1* mice. Body weight over time shown as mean ± SD, n = 8–19 for all time points except for *Npc1*^*-/-*^ mice on UDCA at 13 weeks where n = 4 and 14 weeks where n = 1, # denotes comparison between *Npc1*^*+/+*^ mice untreated and *Npc1*^*+/+*^ mice on UDCA, * denotes comparison between *Npc1*^*-/-*^ mice untreated and *Npc1*^*-/-*^ mice on UDCA, # or * *p—*value < 0.05, ## or ** *p—*value < 0.01, calculated using two-way ANOVA with Bonferroni’s multiple comparison test. (**B**) Effects of UDCA on motor function in *Npc1*^*-/-*^ mice. Total rearing events counted for a period of 5 minutes in *Npc1*^*+/+*^ and *Npc1*^*-/-*^ mice plus or minus UDCA treatment. Data are presented as mean ± SD, n = 8–19. * *p—*value < 0.05, ** *p—*value <0.01, *** *p—*value <0.001, calculated using an unpaired nonparametric test Mann-Whitney test. (**C-F**) Effects of UDCA on tremor in *Npc1*^*-/-*^ mice. Tremor was monitored between 0 and 63 Hz in panels (**C**) and (**D**), and quantified at 43 Hz in (**E**) early symptomatic (9 weeks), (**F**) late symptomatic (11 weeks) time points. 43 Hz is an arbitrary point selected in the high frequency tremor range to allow a simple comparison to be made between treatment groups. The low frequency tremor range overlaps at the low end with grooming activity frequencies and so was not analysed to avoid this as a potential confounder. In both early symptomatic and late symptomatic groups, mice treated with 0.5% UDCA had significantly less tremor than untreated *Npc1*^*-/-*^. Data are presented as mean ± SD, n = 7–19 animals/group, * *p—*value < 0.05, ** *p—*value < 0.01, *** *p—*value < 0.001, **** *p—*value < 0.0001 calculated using one-way Anova with Bonferroni's multiple comparison test.

Monitoring body weight revealed a similar pattern of weight gain and loss in *Npc1*^*-/-*^ mice regardless of treatment. However *Npc1*^*-/-*^ mice treated with UDCA had a generalized reduction in weight that was significant at 5, 6, 9, and 11 weeks compared with the untreated *Npc1*^*-/-*^ mice (*p* value = 0.006, *p* value = 0.032, *p* value = 0.030 and *p* value = 0.002 respectively). This lower body weigh in the UDCA treated mice was also reflected in the *Npc1*^*+/+*^ mice with significant differences noted at 5, 11, and 12 weeks (*p* value = 0.021, *p* value = 0.007 and *p* value = 0.024 respectively). This was an expected result and in agreement with known reduction in body weight observed in other studies with UDCA [43], no significant difference between genders were noted so genders were combined (Fig 5A).

Rearing (a measure of motor coordination) increased in the *Npc1*^*-/-*^ mice treated with UDCA compared to the untreated *Npc1*^*-/-*^ mice. While a high degree of variability was seen for all groups, UDCA-treated *Npc1*^*-/-*^ mice showed marked improvements, which was significant at 5 weeks of age (*p* value = 0.0055) and from 7 to 12 weeks (*p* value = 0.050, *p* value = 0.002, *p* value = 0.013, *p* value = 0.001, *p* value = 0.0007 and *p* value = 0.002 respectively) (Fig 5B). No differences were detected between the *Npc1*^*+/+*^ treated or untreated mice (Fig 5B). *Npc1*^*-/-*^ mice on UDCA also retained motor function longer than untreated *Npc1*^*-/-*^ mice as demonstrated by their ability to rear at 11 weeks, an age at which all untreated *Npc1*^*-/-*^ mice are incapable of rearing (*p* value = 0.0007) (Fig 5B).

Tremor is a disease phenotype observed in the *Npc1*^*-/-*^ mouse model from approximately 7 weeks of age [44]. An age-dependent increase in high frequency tremor (32-53Hz) has been observed in *Npc1* mice, with 43Hz used as an arbitrary point of comparison between treatment groups [12]. *Npc1*^*-/-*^ mice treated with UDCA presented with significantly lower tremor amplitudes than was observed in the untreated *Npc1*^*-/-*^ mice at 9 and 11 weeks of age (Fig 5C–5F). As previously reported [12], untreated *Npc1*^*-/-*^ mice showed significantly higher 43 Hz tremor when compared with untreated *Npc1*^*+/+*^ mice both at 9 and 11 weeks of age (*p* value = 0.0025 and *p* value < 0.00001 (Fig 5E and 5F). Tremor amplitude at 43 Hz was significantly lower at 9 and 11 weeks of age for *Npc1*^*-/-*^ mice treated with UDCA compared with untreated *Npc1*^*-/-*^ mice (*p* value = 0.014 and *p* value = 0.0002) (Fig 5E and 5F). No changes were detected between untreated *Npc1*^*+/+*^ mice and *Npc1*^*+/+*^ mice treated with UDCA at either time point (Fig 5E and 5F).

**Fig 6:**
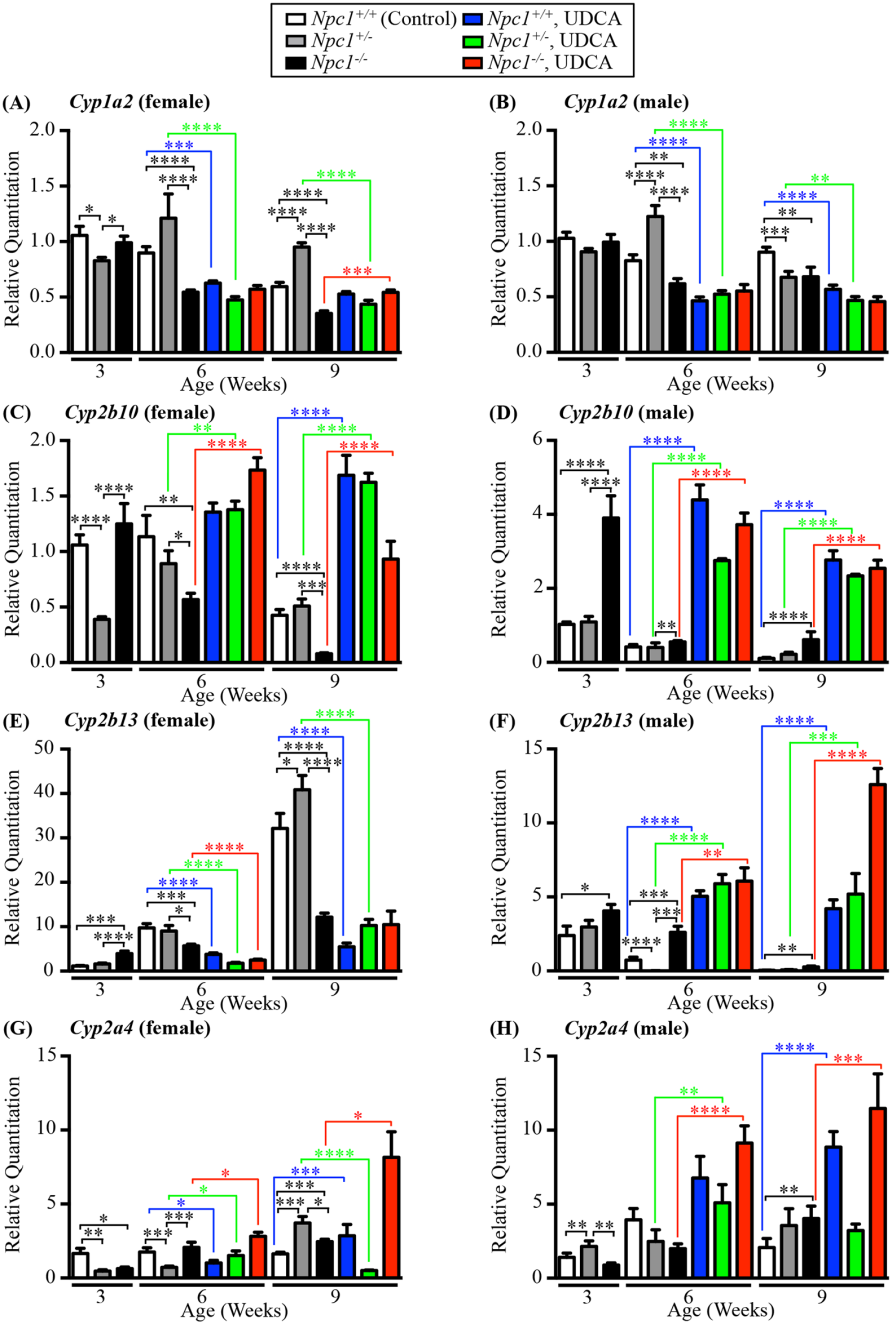
Gene expression analysis pre and post UDCA therapy. Gene expression analysis of 4 CYP genes expression by qPCR in *Npc1*^*+/+*^, *Npc1*^*+/-*^ and *Npc*^*-/-*^ mice (untreated and treated with UDCA) at 3, 6 and 9 weeks old in females and males. White columns indicate *Npc1*^*+/+*^ samples, grey columns indicate *Npc1*^*+/-*^, black columns indicate *Npc1*^*-/-*^, blue columns indicate *Npc1*^*+/+*^ treated with UDCA, green columns indicate *Npc1*^*+/-*^ treated with UDCA and red columns indicate *Npc1*^*-/-*^ mice treated with UDCA. (**A-B**) *Cyp1a2* gene expression. (**C-D**) *Cyp2b10* gene expression. (**E-F**) *Cyp2b13* gene expression. (**G-H**) *Cyp2a4* gene expression. Data are presented as mean ± SEM, n = 3–6. * *p—*value < 0.05, ** *p—*value < 0.01, *** *p—*value < 0.001, **** *p—*value < 0.0001, calculated using an unpaired nonparametric Mann-Whitney test.

Gene expression analysis of a subset of the Cyp genes, *Cyp1a2*, *Cyp2b10*, *Cyp2b13*, and *Cyp2a4* highlight the dynamic nature of these genes and the challenge of fully discerning the exact functional role of each Cyp gene. Basal expression levels of the same genotype and age varied between male and female animals and the manner in which the genders responded to UDCA varied (Fig 6A–6H). *Cyp1a2* gene expression showed little change in the UDCA treated males and females; whilst some values proved to be statistically significant the total fold change was small (Fig 6A and 6B). *Cyp2b10* and *Cyp2a4* expression patterns mirrored the changes in enzymatic activity, with UDCA causing a significant increase in transcript levels (Fig 6C, 6D, 6G and 6H). *Cyp2b13* behaved in the opposite manner based on gender, the females on UDCA exhibiting significantly decreased gene expression, while UDCA-treated males showed significantly increased gene expression (Fig 6E and 6F).

**Fig 7:**
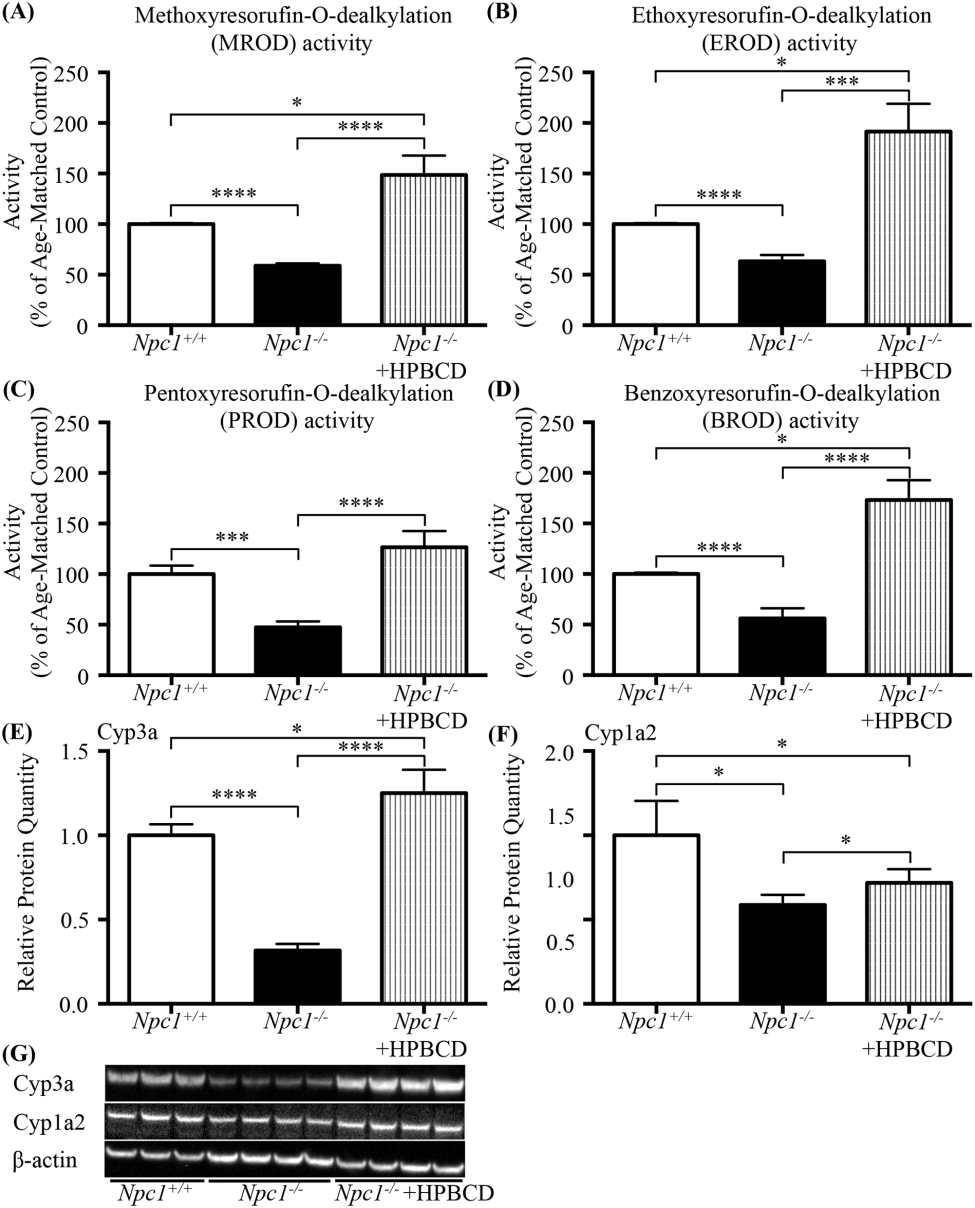
Effect of HPBCD treatment in 7-week old male *Npc1*^*-/-*^ mice. (**A**-**D**) Effects of HPBCD treatment on the activity of CYP450 catalysed reactions in the *Npc1* mouse presented as a function of percent of age-matched control littermates. (**A**) Methoxyresorufin-O-dealkylation (MROD); (**B**) ethoxyresorufin-O-dealkylation (EROD); (**C**) pentoxyresorufin-O-dealkylation (PROD); (**D**) benzoxyresorufin-O-dealkylation (BROD). Data are presented as mean ± SEM, n = 4 (males), * *p—*value < 0.05, *** *p—*value < 0.001, **** *p—*value < 0.0001 calculated using an unpaired nonparametric test Mann-Whitney test. (**E**) Relative protein quantitation of Cyp3a found a significant decrease in *Npc1*^*-/-*^ untreated mice compared *Npc1*^*+/+*^ untreated mice similar to what was seen in Fig 4C. HPBCD treatment increased Cyp3a protein levels in *Npc1*^*-/-*^ mice, both in comparison to the untreated *Npc1*^*+/+*^ or *Npc1*^*-/-*^ mice. (**F**) Relative protein quantitation of Cyp1a2 revealed a significant decrease in *Npc1*^*-/-*^ untreated mice compared *Npc1*^*+/+*^ untreated mice. HPBCD treatment increased *Npc1*^*-/-*^ mice Cyp1a2 protein levels in comparison to untreated *Npc1*^*-/-*^ mice, but did not restore to *Npc1*^*+/+*^ levels. Data are presented as mean ± SD, n = 4. * *p—*value < 0.05, **** *p—*value < 0.0001 calculated using two-tailed, unpaired t test. (**G**) Representative western blot analysis of Cyp3a and Cyp1a2 isolated from 7 week old male *Npc1*^*+/+*^ or *Npc1*^*-/-*^ mouse livers plus or minus HPBCD treatment.

As HPBCD treatment is increasingly being used as a therapeutic modality in the NPC1 patient community we tested the ability of HPBCD to correct the P450 system deficit noted in Fig 4. *Npc1*^-/-^ mice treated with HPBCD had a significant increases in all 4 enzyme activity levels MROD, EROD, PROD, and BROD compared to the *Npc1*^-/-^ untreated mice (P450 activities of *Npc1*^-/-^ mice treated with HPBCD as a % of age-matched control: MROD 148.7 ± 19.16 vs 100 ± 1.04, *p* = 0.037; EROD 191.5 ± 27.59 vs 100 ± 0.92, *p* = 0.039; PROD 126.5 ± 16.16 vs 100 ± 8.54, ns; BROD 173.3 ± 19.47 vs 100 ± 1.27, *p* = 0.012) (% Change in P450 activities levels of *Npc1*^-/-^ mice treated with HPBCD vs untreated *Npc1*^-/-^ mice: MROD 148.7 ± 19.16 vs 58.79 ± 2.31, *p* < 0.0001; EROD 191.5 ± 27.59 vs 63.24 ± 6.15, *p* < 0.0001: PROD activity 126.5 ± 16.16 vs 47.36 ± 5.80, *p* < 0.0001; BROD 173.3 ± 19.47 vs 56.07 ± 10.01, *p* < 0.0001) (Fig 7A–7D). Similar to the UDCA treatment HPBCD restored the protein levels of Cyp3a (*Npc1*^-/-^ HPBCD treated 1.25 ± 0.07 vs *Npc1*^-/-^ untreated 0.32 ± 0.02, *p* < 0.0001; *Npc1*^-/-^ HPBCD treated 1.25 ± 0.07 vs *Npc1*^+/+^ untreated 1.00 ± 0.04, ns) (Fig 7E and 7G) and generated an increase in the protein levels of Cyp1a2 (*Npc1*^-/-^ HPBCD treated 0.72 ± 0.04 vs *Npc1*^-/-^ untreated 0.59 ± 0.03, *p* = 0.041) (Fig 7F and 7G) as noted by Western blot.

**Fig 8:**
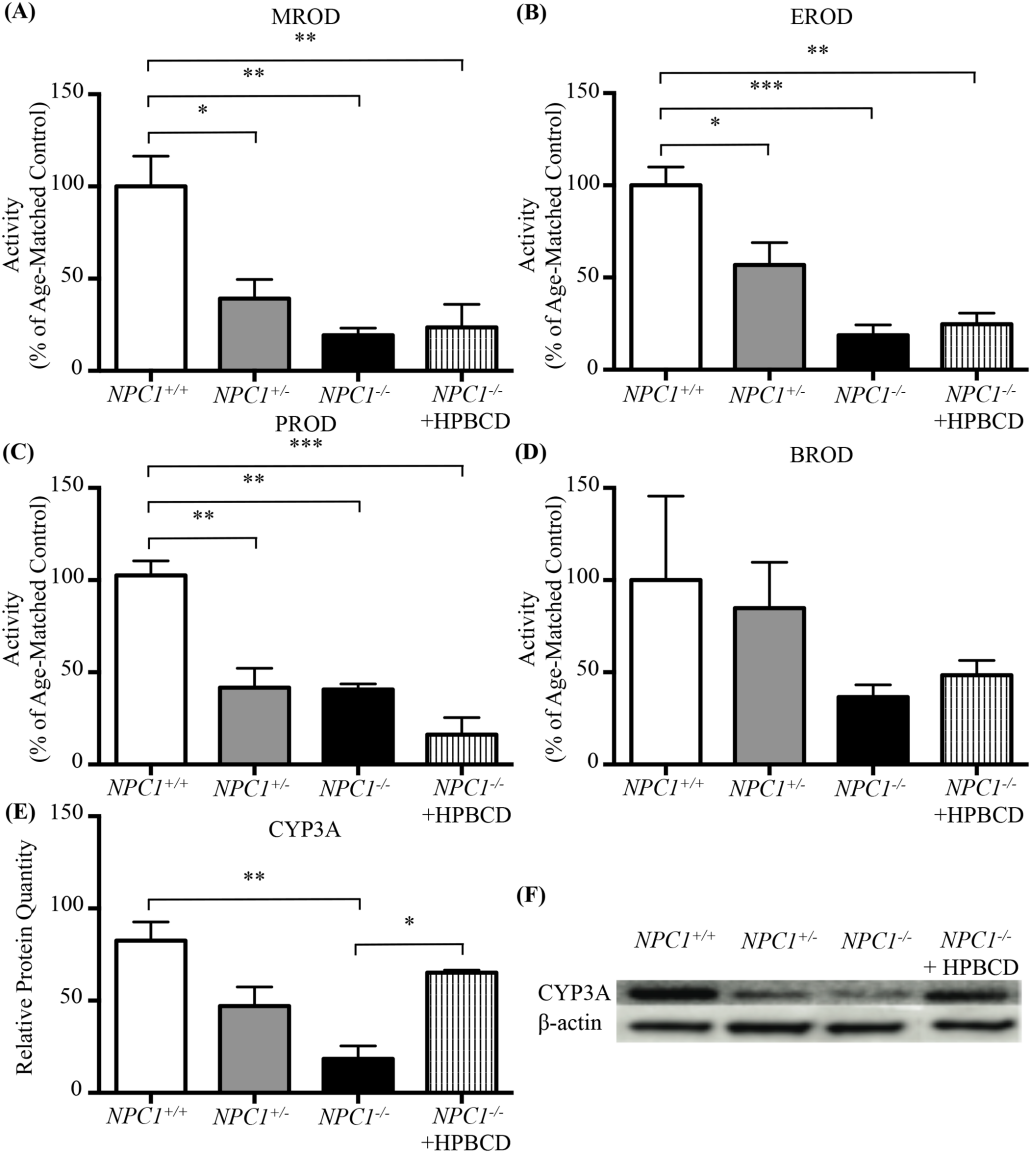
P450 O-dealkylation enzymatic activity in *NPC1*^*+/+*^, *NPC1*^*+/-*^, and *NPC1*^*-/-*^ cats. (**A-D**) Activity of CYP450 catalysed reactions in feline model of NPC1, shown as percent of age-matched controls. Age-matched *NPC1*^*+/+*^ (white), *NPC1*^*+/-*^ (grey) and *NPC1*^*-/-*^ (black) cats were included in addition to HPBCD treated *NPC1*^*-/-*^ cats (striped). (**E**) CYP3A relative protein quantitation from western blot analysis was significantly lower in *NPC1*^*+/-*^, and *NPC1*^*-/-*^ compared to age-matched *NPC1*^*+/+*^ cats when analysed by densitometry. (**F**) Representative western blot analysis of CYP3A in the feline model of NPC1. Age-matched *NPC1*^*+/+*^, *NPC1*^*+/-*^ and *NPC1*^*-/-*^ cats were included in addition to HPBCD treated *NPC1*^*-/-*^ cats. Methoxyresorufin-O-dealkylation (MROD); ethoxyresorufin-O-dealkylation (EROD); pentoxyresorufin-O-dealkylation (PROD); benzoxyresorufin-O-dealkylation (BROD). Data are presented as mean ± SEM, n = 3. Note due to small sample numbers all 3 ages (21, 38, and 59 weeks) HPBCD treated animals were combined for this figure. * *p—*value <0.05, ** *p—*value < 0.01, *** *p—*value < 0.001 calculated using one-way ANOVA test with Tukey’s *post-hoc* test.

To determine if the murine dysfunction in the cytochrome P450 system was species specific or a more global NPC finding we interrogated the *NPC1* feline model. In the *NPC1* feline model, although there was no significant difference in total cytochrome C reductase activity (data not shown), the activities of the specific P450 catalysed dealkylation reactions were significantly lower in *NPC1*^*+/-*^ and *NPC1*^*-/-*^ cats compared to controls (Fig 8A–8C). In liver tissue from *NPC1*^*-/-*^ cats, MROD, EROD, and PROD activity levels (expressed as percentage of age-matched controls) were 19.4 ± 3.9 (*p* = 0.006), 18.7 ± 5.6 (*p* = 0.0008), and 40.6 ± 3.1 (*p* = 0.003) respectively (Fig 8A–8C). However, there was no significant reduction in BROD activity in the *NPC1*^-/-^ cats (36.5 ± 6.6, *p* = 0.38 vs *NPC1*^*+/+*^) (Fig 8D). Whilst activity was reduced to 36.5% of the control mean this did not constitute a significant difference due to high variability in the controls. Consistent with the murine model, decreased activity was also found for some P450 isozymes in heterozygous *NPC1*^*+/-*^ cats MROD, EROD, and PROD activity levels (expressed as percentage of age-matched controls) were 39.2 ± 10.4 (*p* = 0.026 vs *NPC1*^*+/+*^), 56.9 ± 12.1 (*p* = 0.035 vs *NPC1*^*+/+*^), and 41.7 ± 10.4 (*p* = 0.003 vs *NPC1*^*+/+*^) respectively (Fig 8A–8C). BROD showed little or no reduction in *NPC1*^*+/-*^ cats compared to littermate controls (Fig 8D).

Treatment with HPBCD (8000 mg/kg administered subcutaneously every 7 days) did not correct enzyme activity of MROD, EROD, PROD, or BROD in *NPC1*^*-/-*^ cat liver (Fig 8A–8D). Interestingly, treatment with HPBCD did result in the normalization of CYP3A protein expression levels (relative protein quantitation *NPC1*^*-/-*^ 0.15 ± 0.04 vs *NPC1*^*-/-*^ HPBCD treated 0.83 ± 0.006 as a function of age-matched controls, *p* = 0.003) (Fig 8E and 8F). *NPC1*^-/-^ cats treated with HPBCD showed a significant increase in the expression of CYP3A, although this increase in protein did not appear to translate into increased enzyme activity (Fig 8A–8D).

**Fig 9:**
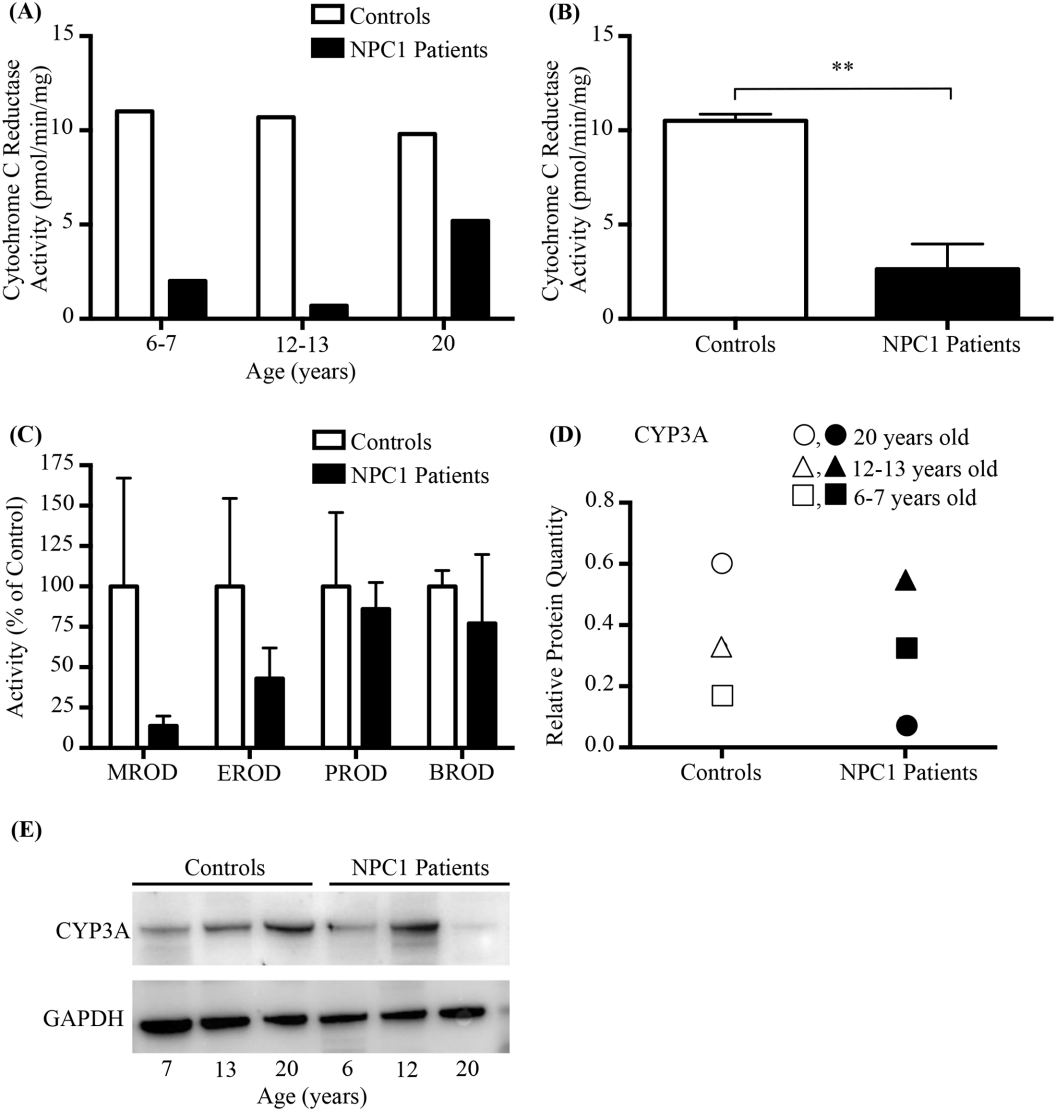
NPC1 patient P450 Analysis. *(A)* Activity of cytochrome C reductase in individual NPC1 patients at various disease stages and age- and gender-matched controls. Data are expressed as pmoles cytochrome C reduced per minute, normalised for microsomal protein content (mg). (**B**) Combined patient samples from panel A, Activity of cytochrome C reductase in liver microsomes from NPC1 patients and age-and gender-matched controls. (**C**) Activity of CYP450 catalysed reactions in NPC1 patients and age-and gender-matched controls. Data are expressed as percentage of age-matched controls. Each individual sample was run in triplicate. (**D**) Relative CYP3A protein quantification from Western Blot analysis in livers from NPC1 patients and controls. The circles indicate the 20 year old NPC1 and control individuals, the triangles the 12–13 year old NPC1 and control individuals, and the squares represent the data from the 6 to 7 year old NPC1 and control individuals. (**E**) Representative western blot analysis of CYP3A in livers of NPC1 patients and their age- and gender-matched controls. Data are presented as mean ± SEM, n = 3. ** *p—*value < 0.01, calculated using two-tailed, unpaired t test.

**Fig 10:**
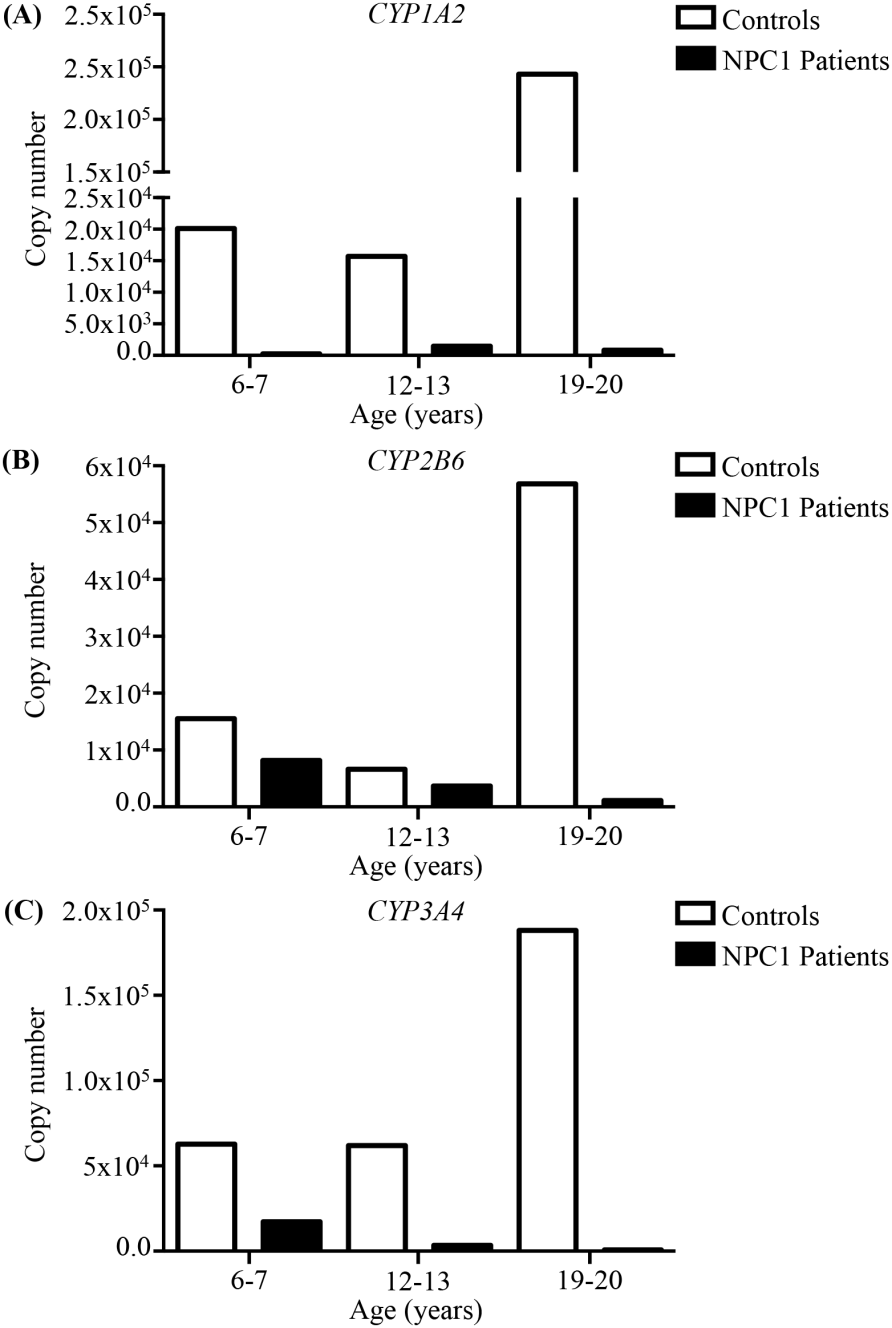
Absolute quantitation of mRNA expression of *CYP1A2*, *CYP2B6*, and *CYP3A4*. Absolute quantitation of mRNA expression of the CYP isozymes in three NPC1 patients (black) and age-matched control (white) livers. (**A**) CYP1A2. (**B**) CYP2B6. (**C**) CYP3A4.

We further extended these studies to NPC1 patients, utilizing post-mortem liver samples, isolated from NPC1 patients and age and gender matched controls. We observed lower cytochrome P450 reductase activity in all three NPC1 individuals (Fig 9A), as well as a significant reduction when combining the data in aggregate (10.5 ± 0.4 (controls) vs 2.6 ± 1.3 (NPC1 patients) pmol/min/mg, *p* = 0.005) (Fig 9B). We also observed decreased MROD and EROD activities, although due to high individual variation these differences were not statistically significant (Fig 9C). No apparent differences were noted in PROD or BROD activities (Fig 9C). Relative protein quantitation analysis of CYP3A revealed no differences in aggregate, however there was an increase in CYP3A in the paediatric NPC1 sample, and a large decrease in the adult NPC1 patient compared to their respective controls (Fig 9D–9E). Absolute quantitation of the *CYP1A2*, *CYP2B6* and *CYP3A4* isozyme mRNA isolated from the human livers revealed a notable reduction in expression of all three genes in the NPC1 patients compared to healthy age and gender matched controls (Fig 10).

## Discussion

We hypothesized that *Npc1*^*-/-*^ mice have defective hepatic drug metabolism based on evidence that (i) liver metabolised compounds caused toxicity when administered at doses well tolerated in other mouse models/strains [12] (Table 1) and (ii) no evidence of toxicity has been noted for renally excreted compounds such as miglustat [45] in *Npc1*^*-/-*^ mice [35]. Since cytochrome P450 enzymes facilitate the clearance of the drugs previously shown to have increased toxicity in *Npc1* mutant mice, this superfamily of enzymes was investigated further in NPC1 animal models. Cross species comparisons are challenging due to interspecies variability, namely the number of isozymes and their specific activities [46]. For example, in human and mouse hepatocytes, P450 isoforms show selectivity for specific substrates [47, 48]. In humans EROD and MROD are mainly catalysed by CYP1A, whilst BROD is catalysed predominantly by CYP3A (CYP1A and CYP2A to lesser extent) and PROD is catalysed by CYP2B [47]. One confounding factor is that the clusters containing the genes encoding Cyp2a, Cyp2b and Cyp3a isozymes are greatly expanded by gene duplication in the mouse compared to human [46, 49] and this close similarity of sequences of Cyp paralogs makes the identification of orthologs difficult. A second confounding issue is there are significant differences between the male and female gender in the rate of drug metabolism and the gene expression of members of the cytochrome P450 family [50], as was observed in Fig 6.

We first investigated the P450 system in the murine model of NPC1 disease. In agreement with our empirical observations, the rates of the individual cytochrome P450-catalysed O-dealkylation reactions were reduced in *Npc1*^*-/-*^ mice at all ages evaluated, consistent with microarray results indicating a global down regulation of the genes in this pathway as early as 1 week of age (S2 Fig) [9]. Notably, impaired P450 activity occurred prior to the onset of clinical manifestations. This reduction in the O-dealkylation reactions was also present in heterozygous *Npc1*^*+/-*^ mice from 6 weeks of age. Consistent with these findings, *Npc1*^-/-^ and *Npc1*^+/-^ mice showed a significant reduction in cytochrome C reductase activity at 9 weeks of age compared to age-matched control mice (S1 Fig). While *Npc1*^-/-^ mice consistently presented with decreased activity and gene expression the *Npc1*^+/-^ mice were more variable in nature. *Npc1*^+/-^ mice presented with decreased activity while gene expression was only decreased in 2 of the genes assayed. Determination of the protein levels of Cyp3a and Cyp1a2 in the *Npc1*^+/-^ mice revealed significant decreases consistent with the enzyme activity assays but divergent of the gene expression data. In addition, it is possible that a greater portion of the 101 known *Cyp* genes are down regulated, but were not assayed here or that the increased expression of a subset of genes is not sufficient to correct the decrease expression of other Cyp genes.

To validate the importance of the observed CYP deficiencies *in vivo*, we administered the CYP3A metabolised drug, midazolam, to *Npc1*^*-/-*^ mice and their wild-type counterparts. Plasma levels of both parent midazolam and CYP3A-mediated midazolam metabolites were increased compared to plasma levels observed in control animals. Increased plasma midazolam levels are consistent with decreased CYP3A4 activity; however, the increase in midazolam metabolites suggests that phase II conjugation of midazolam metabolites (by UGTs) is also impaired in *Npc1* mutant mice. Consistent with this hypothesis, UGT expression was globally decreased similar to decreases in expression of CYPs. Therefore, midazolam metabolism occurs more slowly, but increased exposure to metabolites occurs as a function of a reduction in Phase II conjugating enzymes. As a consequence of impaired drug metabolism, the therapeutic range of prodrugs requiring metabolism to be activated and drugs undergoing hepatic clearance may be altered in NPC1.

To determine if the cytochrome P450 defects are ubiquitous to NPC1 disease as a whole or if this is a species-specific manifestation in the *Npc1*^*-/-*^ mouse model we investigated the feline model of NPC1. Significant dysfunction was noted with reduced activity of O-dealkylation catalyzing enzymes. Protein levels of CYP3A, responsible for about fifty percent of drug metabolism, were decreased in liver from affected cats. HPBCD treatment of the NPC1 cats partially corrected the P450 system by restoring the activities of the O-dealkylation catalyzing enzymes and increased expression of CYP3A. Therefore, it appears HPBCD may partially correct hepatic metabolism deficiencies in addition to improving neurological presentations of NPC1 disease. The mechanism underpinning this selective correction remains unclear, however what is clear is the deregulation and dysfunction of the cytochrome P450 system in NPC1 extends beyond the murine model.

We also measured cytochrome P450 and isozyme activity in post-mortem liver samples from NPC1 patients along with age and gender matched controls. A reduction in activity, consistent with the data from the NPC1 feline and mouse models, was observed. These data suggest that patients with NPC1 may have an increased susceptibility to drug toxicities or drug-drug interactions. A significant limitation of this research is the number of samples available and the manner in which they have been collected. We are only aware of these three NPC1 liver samples in any of the tissue banks worldwide and the variation in the age of the individuals at the time of collection compounds the difficulty in interpreting this data. The NIH has not collected liver biopsies on any NPC1 patients to date. While we are unaware of any direct clinical manifestation or decrease drug tolerances in NPC1 patients, the possibility exists that there could be a subclinical defect in cytochrome P450 system that will need further investigation.

We hypothesised that dysregulation of cytochrome P450 enzymes is linked to the defect in late endosome/lysosome to ER cholesterol efflux. Two pathological processes (S4 Fig) are likely involved: first, an absence of oxysterol activation of LXR, and secondly reduced bile acid synthesis or the synthesis of atypical bile acids as a consequence of cholesterol sequestration in LE/Lys. Enzymatically generated oxysterols are known to be deficient in NPC1 [34]. The reduction in these LXR agonists likely results in the decreased expression of LXR regulated genes, including *CYP7A1*, which catalyses the first step in bile acid biosynthesis [51–54]. This could explain the improvement in the O-dealkylation reactions noted in the feline *NPC1*^*-/-*^ HPBCD treated animals, as HPBCD has been shown to increase bioavailability of endolysosomal sequestered cholesterol, leading to increased oxysterol production [16].

Bile acid biosynthesis is also known to be significantly lower in *Npc1*^*-/-*^ mice compared to control mice when challenged with a high cholesterol diet, likely due to *Npc1*^*-/-*^ mice failing to increase bile acid biosynthesis [55, 56]. Bile acids are endogenous ligands for PXR, which regulates the expression of a large number of Phase I and Phase II drug metabolising enzymes [57]. Thus, it is plausible that one mechanism leading to decreased cytochrome P450 mediated drug metabolism in NPC1 is the decreased enzymatic oxysterol production and subsequent bile acid deficiency. For example, a novel 7-oxo and a high frequency of sulphated and additionally conjugated forms of various cholenoic acids in urine have been detected and are indeed potential biomarkers of the disease [31–33]. We therefore tested whether bile acid deficiency/imbalance contributes to the P450 defect identified in NPC1 disease. *Npc1* mice were supplemented orally from 3 weeks of age with UDCA, which normalized or led to supraphysiological P450 enzyme activities, confirming that bile acid deficiency/imbalance likely participates to the drug metabolism defect in NPC1. Of some concern are the supraphysiological P450 enzyme activity levels reached by all mice receiving UDCA therapy. These elevated activities could lead to increased rates of drug metabolism requiring greater doses to obtain the desired physiological effect or in extreme case may expose patients to greater risk of developing various forms of cancer [58]. However, the dosing used in this mouse model was based on literature values and should be considered to be a proof of concept to probe the mechanism of P450 suppression in NPC1. The dose is approximately 30–40 fold higher than standard clinical dosing in humans. Thus, we are now investigating whether doses more typical of clinical practice can normalize P450 activity in the *NPC1* mouse.

Bile acid supplementation with UDCA, in addition to correcting the P450 defect, also reduced tremor and improved motor function in the *Npc1* mice. The mechanism underlying these observed neurological benefits is not known. One possible explanation is that UDCA ameliorates the mitochondrial defect present in NPC1 [59, 60], resulting in increased adenosine 5’-triphospate synthesis and improved neuromuscular function. Recent studies by Mortiboys *et al*. demonstrated marked improvement in mitochondrial function in both parkin-mutant fibroblasts, and the *LRRK2*^*G2019S*^ fly model of Parkinson disease when treated with UDCA [61, 62]. Of note that mitochondrial dysfunction has been reported in NPC1 disease [59].

The P450 superfamily is known to be differentially regulated in the brain [63]. Secondary effects in the brain within this superfamily have been demonstrated in NPC1 mouse, including the reduction in Cyp46a1 [34]. It is unclear what role CYP gene products in the brain play in the metabolism of xenobiotics that cross the blood-brain barrier [64]. However, evidence exists that variation in the activity of the different CYP family members can result in alterations in xenobiotic responses in the brain. For example, Elbaz *et al*. demonstrated that polymorphisms within *CYP2D6* in farmers exposed to pesticides resulted in an increased risk of developing Parkinson's disease [65]. It is therefore possible that some of the neuropathology in NPC results from reduced xenobiotic metabolism due to lower expression of the P450 system in the brain.

The impaired function of both phase I and II drug metabolism reported in this manuscript has multiple potential preclinical and clinical implications. In preclinical studies, increased toxicity of liver metabolized drugs may occur and drug efficacy may be masked by toxicity due to impaired P450 function. Thus, potentially disease-modifying therapies may have been discounted in previously conducted animal model studies. Adjusting standard doses proportional to the reduced P450 capacity (Table 1) and concurrent utilization of bile acid therapy may mitigate this problem and should be considered for future proof of concept animal model studies utilising drugs that undergo hepatic metabolism. It is interesting to note that some drugs demonstrating efficacy in the NPC1 mouse model, such as curcumin, inhibit P450 enzymes, suggesting that the positive benefits of this weak SERCA antagonist outweigh drug toxicity effects at certain doses. Heterogeneity in response to this natural product has been reported [66, 67]. As the outcome in the *Npc1*^*-/-*^ mouse treated with curcumin may depend on the balance between xenobiotic levels in mouse chow [68, 69] (variable between suppliers and in different countries) and residual P450 function in response to the stage of the disease when the therapy starts, it is possible that this underlies the variation in response to curcumin reported by different laboratories.

Altered P450 metabolism may also explain an intriguing observation regarding liver disease in NPC1. Cholestatic jaundice and hepatic dysfunction is frequently present in neonates with NPC1. Although this may be lethal, it frequently resolves. The normal maturation of both phase I and II metabolism post birth is well described in healthy individuals [70]. Although additional investigation is necessary, it is plausible that impaired phase I and II metabolism in NPC1 in conjunction with the developmental immaturity of these metabolic pathways contributes to the neonatal hepatic manifestations of NPC1.

In conclusion, we have identified a novel drug metabolism defect in NPC1 disease in multiple species (mouse, and feline) as well as providing initial evidence for disturbance in NPC1 patients. These effects can be ameliorated by treatment with bile acid. Treatment with UDCA also decreased tremor severity and improved motor function in the *Npc1* mutant mice suggesting a neurological benefit. These findings suggest that UDCA therapy may be a useful adjunctive therapy in NPC1 disease.

## Materials and Methods

### Animals

#### *Npc1* mouse model

*Npc1* mutant (BALB/cNctr-*Npc1m1N*/J, *Npc1*^-/-^), control (*Npc1*^+/+^) and *Npc1* heterozygous mice (*Npc1*^+/-^), were generated from heterozygote breeding. Genotyping was performed as described by Loftus *et al* [10]. All mice were maintained under a standard 12h light/12h dark cycle with water and food available *ad libitum*. All procedures conducted in Oxford were performed according to the Animals (Scientific Procedures) Act 1986 under a project license (PPL No. 30/2923) from the UK Home Office. All mouse work conducted at the National Institutes of Health conformed to NIH guidelines and was approved by the NICHD Institutional Animal Care and Use Committee.

#### Feline NPC1 Samples

Cats were raised in the animal colony of the School of Veterinary Medicine, University of Pennsylvania, under the guidelines of the National Institutes of Health and US Department of Agriculture for care and use of animals in research. The University of Pennsylvania Institutional Animal Care and Use Committee approved this study. Liver samples from the feline model of NPC1 disease were from age-matched cats (24 weeks old) (*NPC1*^*+/+*^, *NPC1*^*+/-*^ and *NPC1*^*-/-*^) in addition to samples from HPBCD-treated cats (subcutaneous, 8000 mg/kg/ every 7 days beginning at 3 weeks of age), obtained at different ages (21, 38 and 59 weeks old).

### Human Samples

Human tissue was obtained from the National Institute of Child Health and Human Development Brain and Tissue Bank for Developmental Disorders at the University of Maryland, Baltimore, MD, http://medschool.umaryland.edu/BTBank/, contract HHSN275200900011C, Ref. No. N01-HD-9_0011. Liver samples were collected at autopsy from three NPC1 cases patients (UMB# M4002M, UMB# M4003M, UMB# M4004M), and three age- and gender-matched controls (UMB#1500 6 years old had a perforated bowel, haemopericardium, hypoxic ischaemic encephalopathy, secondary to a motor vehicle accident. UMB#662 12 years old died of a traumatic accident with multiple injuries. UMB#1672 died of asphyxia). The National Institute of Child Health and Human Development Institutional Review Board approved human studies.

### Cytochrome P450 Activity Assay

#### Hepatic Microsomal Preparation

Livers were harvested, weighed and minced in ice-cold KCl (1.15%, w/v). Samples were homogenized using a Polytron in ice-cold SET buffer (0.25 M sucrose, 5 mM EDTA and 20 mM Tris-HCl, pH 7.4) in a 1:3 (w/v). Liver homogenates were subjected to differential centrifugation at 4°C as follows: homogenates were first centrifuged at 1,000 g for 20 minutes, the post-nuclear supernatants were spun at 12,000 g for 30 minutes and the resultant post-mitochondrial fractions (supernatants) were centrifuged at 100,000 g for 60 minutes. Microsomal pellets were re-suspended in ice-cold homogenisation (SET) buffer (1:3 w/v). Microsomal protein concentrations were measured using the bicinchoninic acid kit (Sigma-Aldrich, UK) according to the manufacturer’s instructions with bovine serum albumin as the standard.

#### Enzyme Activity

P450 reductase activity was measured spectrophotometrically (Jenway 6305) following the NADPH-dependent reduction of cytochrome C using a commercial cytochrome P450 reductase assay kit (Cytochrome C Reductase (NADPH) Assay Kit, CY0100, Sigma-Aldrich). Microsomal preparations (50 μg protein) were incubated with 950 μL of 36 μM cytochrome C in potassium phosphate buffer (300 mM, pH 7.8) containing 0.1 mM EDTA. The mixture was incubated at 25°C and the reaction initiated by the addition of 100 μL of 0.85 mg/ml NADPH. The rate of the reduction of cytochrome C reaction was followed at 550 nm. Activity was expressed as nmol cytochrome C reduced per min per mg of microsomal protein, using extinction coefficient of 21.1 mM^-1^.cm^-1^. To investigate the individual subfamilies of P450 system enzymes, the following reactions were measured: ethoxyresorufin O-dealkylation (EROD) for CYP1A1, 1A2, methoxyresorufin O-dealkylation (MROD) for CYP1A2, pentoxyresorufin O-dealkylation (PROD) for CYP2B and benzoxyresorufin O-dealkylation (BROD) for 3A (1A, 2A and 2B to a lesser extent) [47, 48, 71, 72]. All samples were assayed in duplicate. Microsomal preparations were incubated with 50 mM Tris–HCl (pH 7.6), MgCl_2_ (25 mM), liver microsomes (0.5 mg protein/ml) and 5μM of the substrate (7-methoxyresorufin, 7-ethoxyresorufin, 7-pentoxyresorufin and 7-benzyloxyresorufin, obtained from Sigma-Aldrich, UK). Reactions were started by adding NADPH (0.25 mM, final concentration) and after 30 minutes of incubation at 37°C, the amount of resorufin formed was spectrofluorimetrically measured with a microplate reader (FLUOstar OPTIMA), using wavelengths for excitation (range 560–570 nm) and emission (range 590–600 nm). The amount of product formed, as an index of enzyme activity, was determined by comparison to a standard curve made with pure resorufin (Sigma-Aldrich) (0–250 pmol) dissolved in DMSO and made up to the final volume with Tris–HCl/MgCl_2_ buffer.

### Western Blotting

Frozen liver tissue was homogenized in RIPA buffer with protease (Roche) and phosphoprotease inhibitors (sodium fluoride, sodium orthovanadate). Protein lysate concentrations were determined using the DC Protein Assay (Bio-Rad). Proteins lysates (15 to 50ug) were resolved in a 4–12% Bis-Tris NuPage gel using the MES buffer system (Invitrogen). Transfer was carried out using the iBlot System (Invitrogen) onto nitrocellulose membranes (human) or transferred onto Hybond-P polyvinylidene difluoride (PVDF) membrane (GE healthcare, Chalfont St. Giles, UK) using the Xcell II Blot Module (Invitrogen) (feline and murine). Membranes were blocked in 5% BSA prepared in Tris-buffered saline containing 0.1% Tween 20 (PBS-T) buffer at 4°C overnight. Incubation with the CYP3A (mouse, Santa Cruz Technologies) primary antibody at a dilution of 1:500 or CYP1A2 (mouse, Santa Cruz Technologies) primary antibody at a dilution of 1:1000 was carried out at 4°C overnight. Washing with PBS-T was carried out prior to incubation with secondary antibody. Incubation for 2 hours with the anti-GAPDH (1:2000 dilution, rabbit) (Cell Signaling Technologies) or monoclonal anti-β-actin antibody-peroxidase conjugate (Sigma) was used as a loading control (at 1:10,000 dilution). Again the membrane was washed with PBS-T. HRP-conjugated anti-mouse and anti-rabbit secondary antibodies were used for developing (1:10,000 dilution, Sigma) or immediately visualized. The Bio-Rad HRP chemiluminescent kit was used for exposure. Detection of bands and quantification was performed using the Bio-Rad Gel Doc Imaging System.

### Gene expression

#### Microarray hybridization and data analysis

Microarray analysis previously reported [9].

#### Real-time PCR

Total RNA (10 μg), from the same set of livers used for the microarray analysis, was reverse-transcribed into cDNA using a High-Capacity cDNA archive kit according to the manufacturer’s instructions (Applied Biosystems). The following Taqman assays were used for the validation of the micro-array data (Applied Biosystems): Cyp2b10 (Mm00456588_mH), Cyp2b13 (Mm00771172_g1), Cyp2c37 (Mm00833845_m1*), Cyp2c40-67-68 (Mm04204172_mH, amplifying the three Cyp2c40, Cyp2c67 and Cyp2c68 mRNA), Cyp2c50 (Mm00663066_gH*), Cyp2c54 (Mm02602271_mH*), Cyp3a16 (Mm00655824_m1*), Cyp3a41a-b (Mm00776855_mH, amplifying both Cyp3a41a and Cyp3a41b mRNA), Cyp3a44 (Mm01703321_mH*), Cyp1a2 (Mm00487227_m1) and Cyp2a4 (Mm00487248_g1). Gapdh gene assay was used as reference (Taqman Rodent GAPDH control reagents, Applied Biosystems). Quantitative real-time PCR was performed in 384-well plates with Applied Biosystems 7900 real-time PCR system, using the same series of samples than for the microarrays. Each sample was analyzed in triplicate, using 50 ng of total cDNA for each reaction. The relative quantification of gene expression was performed with the comparative cycle number measured with the threshold method (C_T_), using the 1-week-old control samples as a reference for quantification. A two-tailed unpaired t-test, with Welch’s correction when necessary was performed to assess the significance of the difference of means between control and *Npc1*^*-/-*^ samples at each age.

#### Absolute quantitation

RNA was extracted after homogenization in TRIzol (Invitrogen) of 50 mg of frozen human liver autopsies. Total RNA (10 μg) was reverse-transcribed into cDNA using a High-Capacity cDNA archive kit according to the manufacturer’s instructions (Applied Biosystems). To perform absolute quantitation on human liver samples, we amplified small fragments by PCR for CYP1A2, CYP2B6 and CYP3A4, using a cDNA library generated from the Huh7 human hepatocyte cell line, and the following primers:

CYP1A2-F ^5’^GAAGCTCCACACCAGCCAT^3’^;

CYP1A2-R ^5’^CCTTGAGCACCCAGAATACC^3’^;

CYP2B6-F ^5’^CATGGAAAAAGAGAAATCCAACG^3’^;

CYP2B6-R ^5’^CTTCTGTGTCCTTGGGGATGA^3’^;

CYP3A4-F ^5’^CAATAACAGTCTTTCCATTCCTC^3’^;

CYP3A4-R ^5’^CATCAATTTCCTCCTGCAG^3’^.

The PCR product was then loaded on a 1% agarose gel for purification of each fragment using the QIAquick gel extraction kit (QIAGEN). DNA concentration was determined using a Qubit fluorometer (Invitrogen). Absolute quantitation PCR was performed in 96-well plates with Applied Biosystems 7300 real-time PCR system, using the following Taqman assays (Applied Biosystems): CYP1A2 (Hs01070369), CYP2B6 (Hs03044634), and CYP3A4 (Hs00430021). Each sample and standard was analyzed in triplicate, using 50ng of total cDNA for each sample, and 10-fold serial dilutions of the standard fragment ranging from 10^6^ to 10^1^ copies. cDNA concentration was then determined for each sample using the standard curve generated by the six standards.

### Functional study

#### Bile Acid Supplementation

*Npc1*^*-/-*^ mice, *Npc1*^*+/-*^ mice, and *Npc1*^*+/+*^ mice (n = 6 per group) were fed either normal chow (RM1 maintenance diet; SDS, London, UK) or normal chow supplemented with ursodeoxycholic acid (0.5%, w/w, Sigma-Aldrich) mixed with powdered diet. Treatment started at weaning (3 weeks of age) and mice were sacrificed at 6 and 9 weeks of age. The dealkylation reaction rates in the liver were determined as described above.

#### HPBCD Mouse Treatment

*Npc1* mutant (BALB/cNctr-*Npc1m1N*/J, *Npc1*^-/-^) mice upon weaning at 3 weeks of age began receiving 4000mg/kg HPBCD weekly delivered by IP injection. All procedures conducted in Oxford were performed according to the Animals (Scientific Procedures) Act 1986 under a project license (PPL No. 30/2923) from the UK Home Office.

#### Mouse behavioural analysis

Motor function and coordination were assessed by observational counting of the total rearing events over a period of 5 minutes (either without support or against the cage wall) every week in the open-field.

Tremor was measured with a commercial tremor monitor (San Diego Instruments) according to manufacturer’s instructions. The mice were monitored weekly for 256s, after a 30s acclimatization period. The tremor monitor was connected to a computer via the National Instruments PCI card and the output (amplitude/time) was analyzed (fast Fourier transform) using LabView software, to give a measurement of power at each frequency (0–63 Hz).

#### Pharmacokinetics of midazolam (MDZ)

Midazolam (MDZ), 1’-OH-midazolam, 4’-OH-midazolam, and lorazepam (LZP) reference standards and ammonium acetate were purchased from Sigma-Aldrich (St. Louis, MO). Methanol, sodium hydroxide, and methyl-t-butyl-ether (MTBE) was purchased from Fisher Scientific (Pittsburgh, PA). Drug-free Balb-C mouse plasma was provided by Innovative Research (Novi, MI). All water used was ultrafiltered (Millipore).

#### Midazolam injections and plasma collection

Tail vein injections of 0.25 mg/kg of midazolam were performed on 8-week-old (± 2 days) *Npc1*^*+/+*^, *Npc1*^*+/-*^ and *Npc1*^*-/-*^ female mice. Blood was collected in heparinized syringes under isofluorane anaesthesia by cardiac puncture, 5min, 30min, 60min and 90min after injection (n = 3 for each genotype and time point). Blood was placed on ice until centrifugation at 1,200g for 5 min. The collected plasma was flash frozen on dry ice, and stored at -80°C.

#### Measurement of midazolam and its metabolites by LC/MS

Calibration standards of midazolam (MDZ) and its CYP3A4-mediated metabolites, both 1’- and 4’-hydroxy-midazolam, were prepared together in ethanol at concentrations of 10/1/1, 20/2/2, 50/5/5, 100/10/10, 500/50/50, 750/75/75, and 1500/150/150 for parent midazolam, 1’-OH, and 4’-OH midazolam, respectively. S3 Fig provides the chemical structures of midazolam, both metabolites, and the internal standard lorazepam. Quality control standards were also prepared in batch in mouse plasma at concentrations of 30/3/3, 400/40/40, and 1000/100/100 for MDZ, 1’-OH-, and 4’-OH-MDZ, respectively. Midazolam and its metabolites were extracted from 100 μL of mouse plasma by the addition of 200 μL of 2 mM sodium hydroxide to precipitate plasma proteins, followed by 1 ml of 4000 ng/ml LZP (internal standard; IS) in MTBE. Samples were vortexed, centrifuged, dried down under a stream of desiccated air, and reconstituted with 100μL of (45/55, v/v) 10 mM ammonium acetate/methanol. Samples were again vortexed and transferred to a mini-centrifuge tube for further centrifugation, before the supernatant was transferred to a glass Agilent HPLC vial and injected onto an LC/MS system. A gradient elution was used to separate midazolam, the metabolites, and lorazepam, and consisted of an initial mobile phase of (45/55, v/v) 10 mM ammonium acetate/methanol that was increased linearly to 75% methanol over 8 min, then immediately back to 55% B for the remainder of the 10 min run. The column used was a Zorbax SB-C18 (2.1x75mm, 3.5μm), flow rate was 0.35 ml/min, column temperature was 35°C, and the injection volume was 10 μL. Midazolam, metabolites, and IS were detected using single ion monitoring (SIM) by mass spectrometry in the positive electrospray ionization mode, with one SIM for MDZ (*m/z* 326.1), one for both metabolites (*m/z* 342.1), and the third for LZP (*m/z* 321.2). Because both metabolites had the same molecular weight, thus same molecular ion ([M-H]^+^), the chromatography was able to separate each metabolite for individual identification; however both peak areas were integrated together as one for simplicity. Other mass spectrometric conditions were: capillary voltage 2500V, fragmentor 100V, drying gas temperature 330°C, and a drying gas flow of 5 L/min. A typical LC-MS chromatogram is depicted in S3 Fig.

### Statistical Analysis

A two-tailed, unpaired, nonparametric (Mann-Whitney) or student t-test was performed to compare the residual enzyme activities or protein expression of the different groups. One-way ANOVA with Tukey’s post-hoc test was used to compare all sets of data where applicable. A two-tailed, unpaired t test, with Welch’s correction for heteroscedasticity when necessary, was performed to determine the significance of the difference in cytochrome P450 gene expression levels between *Npc1*^*+/+*^, *Npc1*^*+/-*^ and *Npc1*^*-/-*^ mice at each age. Behavioural studies were analysed by using one-way/two-way ANOVA with Bonferroni’s multiple comparison test or an unpaired nonparametric test (Mann-Whitney) as appropriate. Statistical analysis was performed with GraphPad Prism.

For the midazolam functional study, using Bailer’s Method for destructive sampling, exposures (AUC) for both parent midazolam and metabolites, were calculated and compared between the genotypes. The mean (n = 3) plasma concentrations of each analyte (midazolam or metabolites) at each time point were averaged and a mean AUC ± standard deviation was calculated for each genotype (total n = 12; 3 mice at 4 time points). The AUC values were then subjected to both a one-way ANOVA as well as unpaired Student’s t-tests (with Welch’s correction) for direct comparisons between genotypes.

## Supporting Information

S1 Fig Activity of Cytochrome C reductase in mouse model of NPC1 at 9 weeks of age. Data are expressed as pmoles cytochrome C reduced per minutes normalised for microsomal protein content (mg). Data are presented as mean ± SEM, n = 5, * *p*—value <0.05, ** *p*—value < 0.01, calculated using two-tailed unpaired nonparametric Mann-Whitney test.
(TIF)

S2 Fig Microarray data and subsequent qPCR validation of 9 cytochrome P450 genes. Microarray data and subsequent qPCR validation of 9 cytochrome P450 gene expression performed at 1, 3, 5, 7, 9 and 11 weeks old in the *Npc1* mouse model. qPCR (left axis) and array data (right axis) are presented on the same graph for comparison. Control mice samples figure in white, *Npc1*^*-/-*^ mice in black, n = 4. Only females were used. A two-tailed, unpaired t test, with Welch’s correction when necessary, was performed to determine the significance of the difference in means between control and mutant mice at each age: * *p*—value<0.05; ** *p*—value<0.001; *** *p*—value < 0.0001. Result of the ANOVA test is shown for the array data.
(TIF)

S3 Fig (**A**) Structural formulae of midazolam (1), its metabolites 1’-hydroxy-midazolam (2) and 4’-hydroxy-midazolam (3) and the internal standard Lorazepam (4). (**B**) LC-MS chromatogram of midazolam (top; 5.7 min), 4’-OH and 1’-OH-midazolam (middle; 3.7 and 4.0, respectively), and lorazepam (IS; bottom; 2.9 min).
(TIF)

S4 Fig Schematic of the proposed pathway depicting the effect of NPC mutations on oxysterols, bile acids and P450 system. In the presence of a functional NPC1 and NPC2 protein, LDL-derived cholesterol is trafficked out of the lysosome where it can be converted to side chain oxysterols. Side chain oxysterols activate LXR target genes, including bile acid synthesis enzymes. Bile acids in turn activate other nuclear receptors FXR, CAR and PXR. These nuclear receptors stimulate the transcription of cytochrome P450 enzymes. However, NPC1/NPC2 dysfunction (changes in red) reduces side chain oxysterols and subsequently down regulates the expression levels of downstream enzymes and nuclear receptors, resulting in low expression levels of the xenobiotic metabolizing enzymes and transporter proteins.
(TIF)

S1 Table Micro array analysis of the 62 differentially expressed cytochromes P450 genes. List of the 62 genes encoding cytochromes P450, with a modified expression in 1, 3, 5, 7, 9, and 11-week-old *Npc1*^*-/-*^ mice compared to their control littermates. 42 of these genes belong to the subfamilies 1 to 3 mainly responsible for drug metabolism. FC: fold-change, ns: not significant.
(DOCX)

S2 Table Micro array analysis of the 15 differentially expressed UGT genes. List of the 15 genes encoding UGT, with a modified expression in 1, 3, 5, 7, 9, and 11-week-old ***Npc1***^***-/-***^ mice compared to their control littermates. FC: fold-change, ns: not significant.
(DOCX)

## Abbreviations

GSL: glycosphingolipids
NPC: Niemann-Pick type C
MROD: methoxyresorufin-O-dealkylation
EROD: ethoxyresorufin-O-dealkylation
BROD: benzoxyresorufin-O-dealkylation
PROD: pentoxyresorufin-O-dealkylation
HPBCD: 2-hydroxypropyl-β-cyclodextrin
LXR: liver X receptor
PXR: pregnane X receptor
TNF-α: tumour necrosis factor-α
ER: Endoplasmic reticulum
FXR: farnesoid X receptor
SERCA: sarco/endoplasmic reticulum Ca^2+^-ATPase
UDCA: Ursodeoxycholic acid
UGT: UDP-glucuronosyltransferase
LC/MS: liquid chromatography/mass spectrometry
AUC: area under the plasma concentration vs time curve
