# Supplementary figures and images for: Defective Cytochrome P450-Catalysed Drug Metabolism in Niemann-Pick Type C Disease
Source: PLoS One. 2016 Mar 28;11(3):e0152007. doi: 10.1371/journal.pone.0152007 (PMC4809520; doi:10.1371/journal.pone.0152007)

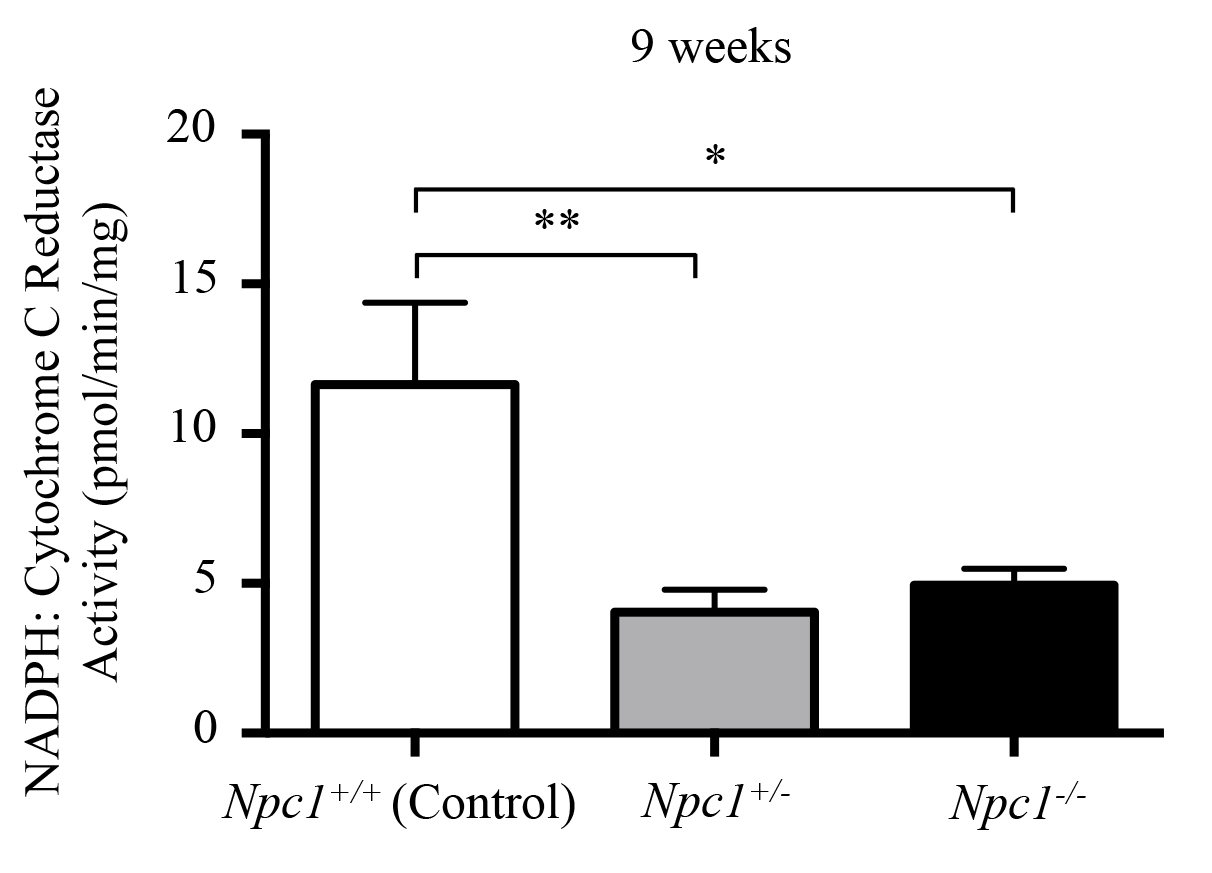

Supplement: S1 Fig — Data are expressed as pmoles cytochrome C reduced per minutes normalised for microsomal protein content (mg). Data are presented as mean ± SEM, n = 5, * p—value <0.05, ** p—value < 0.01, calculated using two-tailed unpaired nonparametric Mann-Whitney test. (TIF) [file pone.0152007.s001.tif]

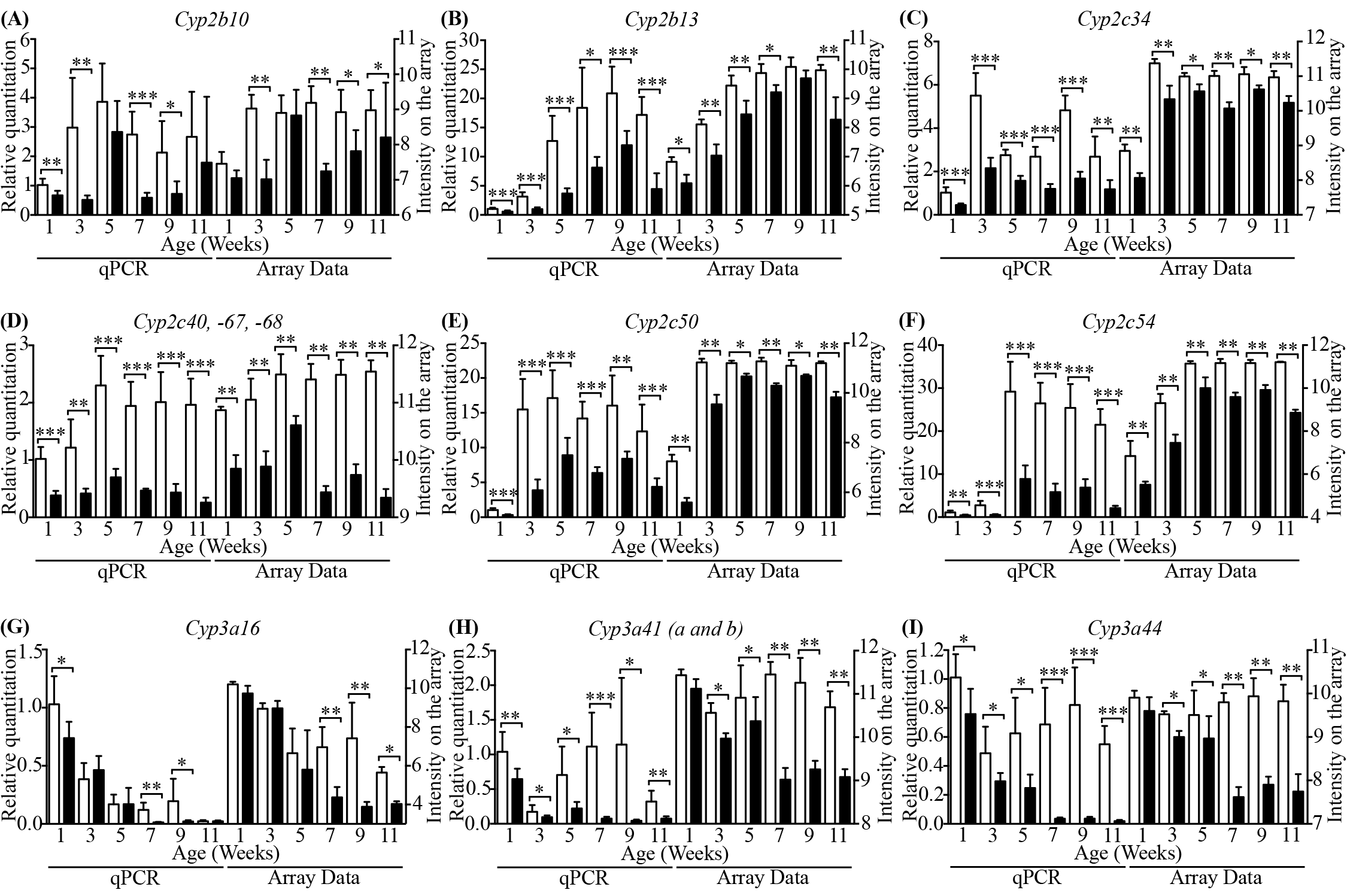

Supplement: S2 Fig — Microarray data and subsequent qPCR validation of 9 cytochrome P450 gene expression performed at 1, 3, 5, 7, 9 and 11 weeks old in the Npc1 mouse model. qPCR (left axis) and array data (right axis) are presented on the same graph for comparison. Control mice samples figure in white, Npc1-/- mice in black, n = 4. Only females were used. A two-tailed, unpaired t test, with Welch’s correction when necessary, was performed to determine the significance of the difference in means between control and mutant mice at each age: * p—value<0.05; ** p—value<0.001; *** p—value < 0.0001. Result of the ANOVA test is shown for the array data. (TIF) [file pone.0152007.s002.tif]

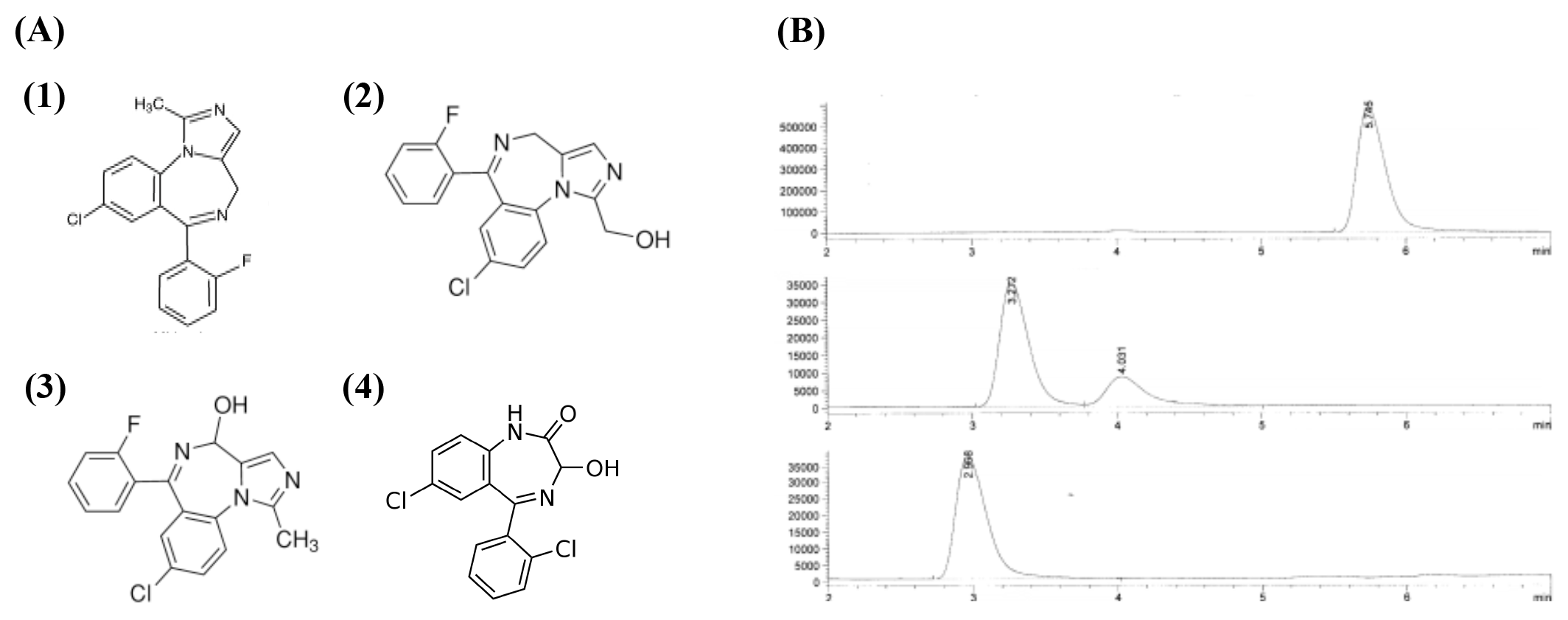

Supplement: S3 Fig — (A) Structural formulae of midazolam (1), its metabolites 1’-hydroxy-midazolam (2) and 4’-hydroxy-midazolam (3) and the internal standard Lorazepam (4). (B) LC-MS chromatogram of midazolam (top; 5.7 min), 4’-OH and 1’-OH-midazolam (middle; 3.7 and 4.0, respectively), and lorazepam (IS; bottom; 2.9 min). (TIF) [file pone.0152007.s003.tif]

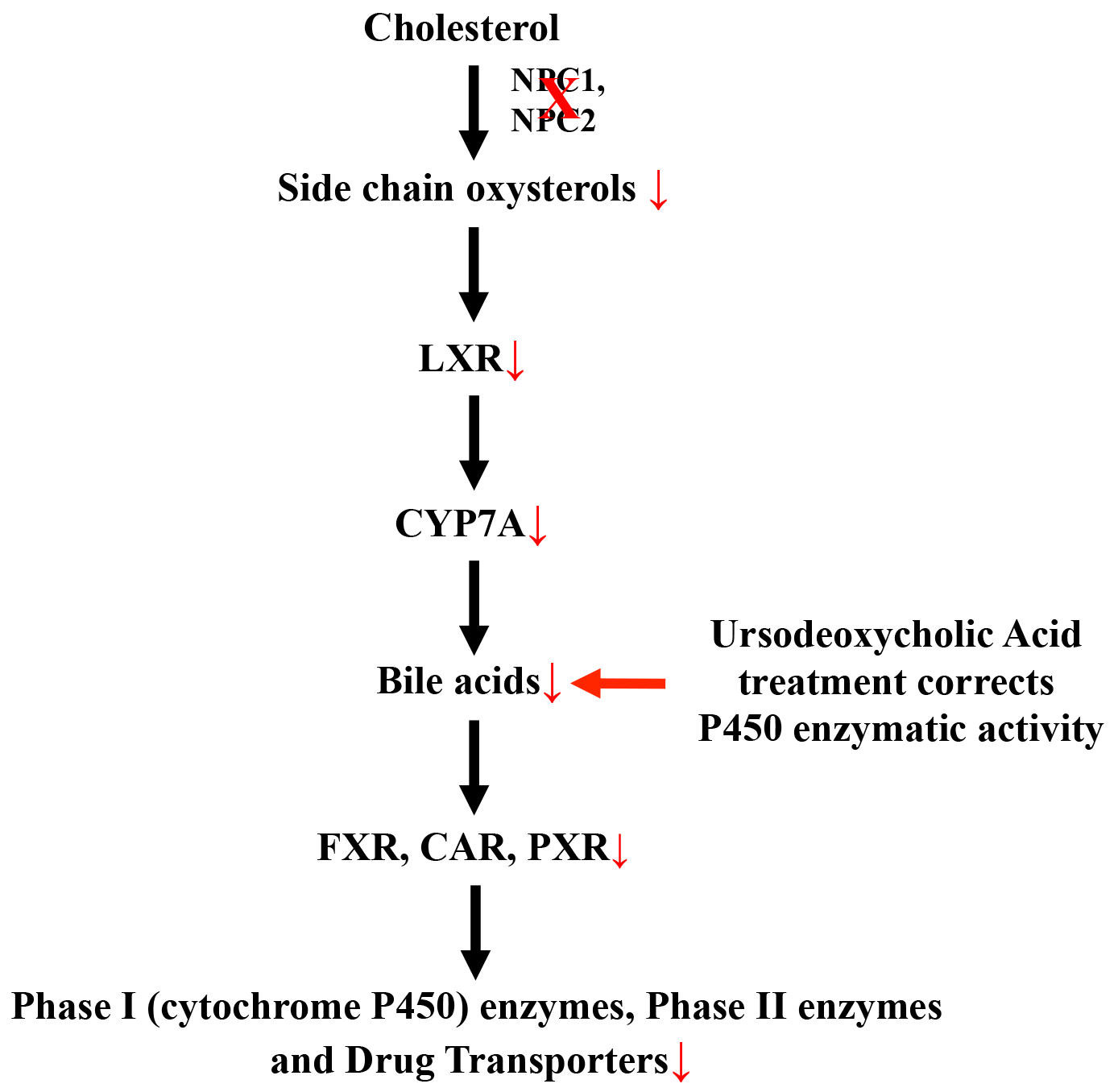

Supplement: S4 Fig — In the presence of a functional NPC1 and NPC2 protein, LDL-derived cholesterol is trafficked out of the lysosome where it can be converted to side chain oxysterols. Side chain oxysterols activate LXR target genes, including bile acid synthesis enzymes. Bile acids in turn activate other nuclear receptors FXR, CAR and PXR. These nuclear receptors stimulate the transcription of cytochrome P450 enzymes. However, NPC1/NPC2 dysfunction (changes in red) reduces side chain oxysterols and subsequently down regulates the expression levels of downstream enzymes and nuclear receptors, resulting in low expression levels of the xenobiotic metabolizing enzymes and transporter proteins. (TIF) [file pone.0152007.s004.tif]
