# Supplementary material for: Defective Cytochrome P450-Catalysed Drug Metabolism in Niemann-Pick Type C Disease
Source: PLoS One. 2016 Mar 28;11(3):e0152007. doi: 10.1371/journal.pone.0152007 (PMC4809520; doi:10.1371/journal.pone.0152007)
Supplement: S1 Table — List of the 62 genes encoding cytochromes P450, with a modified expression in 1, 3, 5, 7, 9, and 11-week-old Npc1-/- mice compared to their control littermates. 42 of these genes belong to the subfamilies 1 to 3 mainly responsible for drug metabolism. FC: fold-change, ns: not significant. (DOCX) [file pone.0152007.s005.docx]

| **Gene Symbol** | **1 week** | | **3 weeks** | | **5 weeks** | | **7 weeks** | | **9 weeks** | | **11 weeks** | |
| --- | --- | --- | --- | --- | --- | --- | --- | --- | --- | --- | --- | --- |
|  | p-value | FC | p-value | FC | p-value | FC | p-value | FC | p-value | FC | p-value | FC |
| Cyp1a1 | ns | ns | ns | ns | ns | ns | ns | ns | 3.03E-02 | -1.31 | 2.51E-02 | 1.32 |
| Cyp1b1 | ns | ns | ns | ns | ns | ns | ns | ns | 5.50E-04 | 1.36 | 4.71E-07 | 1.61 |
| Cyp2a4 | 1.92E-04 | 1.85 | 9.85E-03 | -1.51 | 2.16E-02 | -1.44 | ns | ns | ns | ns | ns | ns |
| Cyp2a5 | ns | ns | 9.52E-03 | -1.44 | ns | ns | ns | ns | ns | ns | ns | ns |
| Cyp2a12 | 2.48E-02 | -1.31 | 3.94E-03 | -1.42 | ns | ns | 4.07E-02 | -1.28 | ns | ns | 1.88E-02 | -1.33 |
| Cyp2b9 | 1.05E-03 | -1.35 | 7.65E-03 | -1.27 | 1.83E-04 | -1.41 | 7.00E-07 | -1.62 | 1.78E-05 | -1.50 | 4.79E-15 | -2.46 |
| Cyp2b10 | ns | ns | 1.56E-06 | -4.03 | ns | ns | 2.97E-06 | -3.85 | 4.60E-03 | -2.16 | 4.46E-02 | -1.71 |
| Cyp2b13 | 1.53E-03 | -1.67 | 1.00E-05 | -2.10 | 3.62E-05 | -1.99 | 4.18E-03 | -1.58 | ns | ns | 2.18E-10 | -3.23 |
| Cyp2b19 | ns | ns | 6.86E-03 | -1.22 | 2.27E-04 | -1.33 | 2.01E-02 | -1.19 | 4.74E-02 | -1.16 | ns | ns |
| Cyp2b23 | ns | ns | ns | ns | ns | ns | 3.92E-02 | -1.21 | 2.97E-03 | -1.32 | ns | ns |
| Cyp2c29 | 1.04E-06 | -1.64 | 1.03E-08 | -1.83 | ns | ns | 6.70E-04 | -1.39 | 2.36E-04 | -1.43 | 8.00E-12 | -2.16 |
| Cyp2c37 | 1.21E-06 | -1.72 | 1.26E-09 | -2.05 | 4.04E-03 | -1.35 | 1.90E-08 | -1.91 | 3.49E-03 | -1.36 | 3.73E-06 | -1.66 |
| Cyp2c38 | ns | ns | 2.83E-12 | -4.52 | 2.98E-03 | -1.71 | 7.32E-03 | -1.62 | ns | ns | ns | ns |
| Cyp2c39 | 2.76E-08 | -1.72 | 3.28E-08 | -1.72 | ns | ns | ns | ns | ns | ns | ns | ns |
| Cyp2c40 | 3.15E-07 | -2.03 | 1.44E-08 | -2.24 | 5.47E-06 | -1.85 | 3.91E-16 | -3.91 | 4.09E-14 | -3.36 | 3.48E-18 | -4.60 |
| Cyp2c44 | 1.45E-03 | -1.46 | ns | ns | ns | ns | 4.14E-05 | -1.66 | 3.65E-06 | -1.79 | 2.28E-17 | -3.86 |
| Cyp2c50 | 1.60E-15 | -3.15 | 7.03E-16 | -3.22 | 1.11E-03 | -1.44 | 3.45E-08 | -1.97 | 1.04E-02 | -1.33 | 1.46E-12 | -2.60 |
| Cyp2c54 | 1.03E-07 | -2.53 | 1.26E-11 | -3.60 | 2.98E-06 | -2.20 | 1.55E-09 | -2.98 | 4.26E-07 | -2.39 | 1.91E-15 | -5.12 |
| Cyp2c55 | ns | ns | 2.30E-02 | -1.63 | 2.61E-03 | 1.92 | ns | ns | ns | ns | ns | ns |
| Cyp2c67 | 8.05E-09 | -2.49 | 2.81E-07 | -2.20 | 7.74E-06 | -1.95 | 1.85E-10 | -2.84 | 1.18E-06 | -2.09 | 7.81E-15 | -4.05 |
| Cyp2c68 | 1.65E-03 | -1.48 | 3.44E-05 | -1.71 | 9.91E-04 | -1.51 | 8.27E-11 | -2.57 | 7.29E-04 | -1.53 | 1.80E-09 | -2.34 |
| Cyp2c70 | 1.23E-12 | -2.07 | 1.31E-09 | -1.79 | ns | ns | ns | ns | ns | ns | ns | ns |
| Cyp2d9 | ns | ns | 1.64E-05 | -1.56 | 7.68E-04 | -1.40 | 5.66E-04 | -1.42 | ns | ns | ns | ns |
| Cyp2d10 | ns | ns | 4.01E-07 | -1.41 | 1.30E-02 | -1.17 | 1.30E-07 | -1.43 | 4.56E-05 | -1.30 | 1.86E-09 | -1.53 |
| Cyp2d11 | ns | ns | ns | ns | ns | ns | 1.07E-02 | -1.33 | ns | ns | 9.08E-03 | -1.34 |
| Cyp2d13 | 2.46E-05 | -1.72 | 9.52E-04 | -1.51 | 1.23E-03 | -1.50 | 1.44E-05 | -1.75 | 6.74E-03 | -1.40 | 1.26E-15 | -3.59 |
| Cyp2d34 | ns | ns | 1.87E-04 | -1.34 | 1.68E-02 | -1.20 | ns | ns | ns | ns | ns | ns |
| Cyp2d37-ps | ns | ns | 2.77E-03 | -1.55 | 4.03E-02 | -1.34 | 3.58E-03 | -1.53 | ns | ns | 1.00E-10 | -3.00 |
| Cyp2d40 | 3.06E-04 | -1.72 | 4.78E-03 | -1.52 | 1.15E-03 | -1.62 | 1.68E-08 | -2.52 | 1.34E-04 | -1.78 | 1.30E-14 | -4.21 |
| Cyp2e1 | 3.67E-06 | -1.32 | 4.07E-03 | -1.17 | 2.12E-02 | -1.14 | 1.93E-02 | -1.14 | ns | ns | 2.55E-02 | -1.13 |
| Cyp2f2 | ns | ns | 4.69E-06 | -1.63 | 1.43E-02 | -1.28 | 1.91E-03 | -1.37 | 8.70E-07 | -1.70 | 4.19E-14 | -2.59 |
| Cyp2g1 | 1.58E-02 | -1.29 | 7.84E-03 | -1.33 | 2.89E-04 | -1.49 | 3.96E-06 | -1.69 | 1.17E-02 | -1.31 | 1.31E-02 | -1.30 |
| Cyp2j5 | 6.82E-09 | -1.70 | 1.92E-12 | -2.01 | 1.13E-02 | -1.23 | 7.78E-12 | -1.95 | 3.23E-13 | -2.08 | 1.57E-28 | -4.97 |
| Cyp2j6 | ns | ns | 4.06E-03 | -1.20 | 9.44E-03 | 1.18 | 1.99E-07 | -1.44 | 4.17E-02 | -1.14 | 5.16E-03 | -1.20 |
| Cyp2j9 | 2.85E-03 | -1.69 | 3.29E-02 | -1.44 | ns | ns | ns | ns | 8.17E-03 | -1.59 | ns | ns |
| Cyp2r1 | ns | ns | 3.55E-02 | -1.33 | 1.20E-02 | 1.41 | 3.04E-01 | -1.15 | 2.83E-01 | -1.15 | 1.59E-02 | -1.39 |
| Cyp3a11 | ns | ns | ns | ns | ns | ns | ns | ns | 2.98E-02 | 1.36 | ns | ns |
| Cyp3a13 | 1.63E-02 | -1.42 | ns | ns | ns | ns | 7.63E-03 | -1.48 | ns | ns | ns | ns |
| Cyp3a16 | ns | ns | ns | ns | ns | ns | 3.11E-05 | -6.02 | 7.12E-08 | -11.55 | 6.48E-03 | -3.08 |
| Cyp3a41a | ns | ns | 2.62E-02 | -1.50 | 4.87E-02 | -1.43 | 6.67E-13 | -5.03 | 5.62E-10 | -3.71 | 1.48E-07 | -2.88 |
| Cyp3a41b | ns | ns | 3.07E-02 | -1.52 | 4.05E-02 | -1.48 | 2.96E-13 | -5.78 | 1.11E-10 | -4.34 | 4.38E-08 | -3.26 |
| Cyp3a44 | ns | ns | 1.64E-02 | -1.44 | 1.33E-02 | -1.45 | 6.01E-15 | -4.54 | 8.36E-14 | -4.10 | 4.12E-14 | -4.22 |
| Cyp4a10 | ns | ns | 4.65E-02 | 1.55 | ns | ns | ns | ns | ns | ns | ns | ns |
| Cyp4a12a | 3.27E-04 | 2.37 | ns | ns | 9.17E-04 | -2.20 | ns | ns | ns | ns | ns | ns |
| Cyp4a12b | 2.65E-02 | 1.52 | ns | ns | 2.08E-02 | -1.55 | ns | ns | ns | ns | ns | ns |
| Cyp4a14 | ns | ns | ns | ns | ns | ns | ns | ns | 8.73E-03 | 2.00 | 3.31E-04 | 2.65 |
| Cyp4a31 | ns | ns | ns | ns | ns | ns | ns | ns | 4.98E-02 | 1.55 | ns | ns |
| Cyp4a32 | ns | ns | 2.78E-02 | 1.50 | ns | ns | 2.11E-02 | -1.53 | ns | ns | ns | ns |
| Cyp4f13 | 5.17E-03 | -1.19 | 2.57E-06 | -1.36 | 4.16E-03 | -1.19 | 1.22E-07 | -1.43 | 2.86E-07 | -1.41 | 1.56E-07 | -1.42 |
| Cyp4f14 | 2.40E-04 | -1.48 | 4.13E-15 | -2.85 | 6.41E-05 | -1.54 | 1.73E-13 | -2.58 | 4.35E-13 | -2.52 | 1.65E-25 | -5.90 |
| Cyp4f15 | 1.66E-02 | -1.19 | 1.04E-03 | -1.28 | 5.77E-03 | -1.23 | 1.96E-07 | -1.53 | 5.76E-05 | -1.37 | 5.13E-14 | -2.02 |
| Cyp4f16 | 4.99E-02 | 1.18 | ns | ns | 1.90E-03 | 1.32 | 1.70E-05 | 1.49 | 4.37E-04 | 1.37 | 7.01E-05 | 1.44 |
| Cyp4f40 | ns | ns | 1.54E-02 | -1.14 | ns | ns | 8.37E-07 | -1.32 | ns | ns | 4.42E-05 | -1.25 |
| Cyp4v3 | ns | ns | 2.52E-03 | -1.22 | ns | ns | 4.62E-07 | -1.43 | 1.56E-02 | -1.17 | 6.61E-12 | -1.71 |
| Cyp7b1 | ns | ns | 8.03E-03 | -1.36 | 2.41E-06 | -1.81 | 6.57E-11 | -2.46 | 1.92E-11 | -2.55 | 1.07E-18 | -4.24 |
| Cyp8b1 | ns | ns | ns | ns | 3.99E-02 | 1.50 | 4.22E-02 | -1.49 | 1.74E-02 | -1.60 | 1.75E-06 | -2.76 |
| Cyp17a1 | ns | ns | ns | ns | ns | ns | 2.36E-02 | -1.90 | ns | ns | ns | ns |
| Cyp20a1 | ns | ns | 2.72E-02 | 1.18 | 1.86E-03 | 1.27 | 2.58E-02 | 1.18 | 5.22E-05 | 1.38 | 7.91E-04 | 1.30 |
| Cyp26a1 | ns | ns | ns | ns | 6.59E-04 | -2.88 | 2.38E-02 | -1.98 | 8.43E-04 | -2.81 | 4.62E-02 | -1.82 |
| Cyp27a1 | ns | ns | 3.12E-04 | -1.33 | 5.80E-05 | -1.38 | 2.66E-08 | -1.60 | 8.00E-07 | -1.50 | 4.55E-03 | -1.24 |
| Cyp39a1 | ns | ns | ns | ns | 2.87E-04 | 1.71 | 2.45E-02 | 1.38 | 2.61E-05 | 1.88 | 2.40E-10 | 2.86 |
| Cyp51 | 5.94E-05 | 2.52 | 3.88E-06 | 2.97 | 1.53E-04 | 2.37  2.37 | 3.67E-02 | 1.58 | ns | ns | ns | ns |

S1 Table. Micro array analysis of the 62 differentially expressed cytochromes P450 genes. List of the 62 genes encoding cytochromes P450, with a modified expression in 1, 3, 5, 7, 9, and 11-week-old *Npc1^-/-^* mice compared to their control littermates. 42 of these genes belong to the subfamilies 1 to 3 mainly responsible for drug metabolism. FC: fold-change, ns: not significant.
