# Supplementary material for: Defective Cytochrome P450-Catalysed Drug Metabolism in Niemann-Pick Type C Disease
Source: PLoS One. 2016 Mar 28;11(3):e0152007. doi: 10.1371/journal.pone.0152007 (PMC4809520; doi:10.1371/journal.pone.0152007)
Supplement: S2 Table — List of the 15 genes encoding UGT, with a modified expression in 1, 3, 5, 7, 9, and 11-week-old Npc1-/- mice compared to their control littermates. FC: fold-change, ns: not significant. (DOCX) [file pone.0152007.s006.docx]

| **Gene Symbol** | **1 week** | | **3 weeks** | | **5 weeks** | | **7 weeks** | | **9 weeks** | | **11 weeks** | |
| --- | --- | --- | --- | --- | --- | --- | --- | --- | --- | --- | --- | --- |
|  | p-value | FC | p-value | FC | p-value | FC | p-value | FC | p-value | FC | p-value | FC |
| Ugt1a9 | ns | ns | ns | ns | 0.018 | -1.23 | ns | ns | 0.008 | -1.26 | 0.029 | -1.21 |
| Ugt2a1 | ns | ns | ns | ns | 0.04 | -1.11 | ns | ns | ns | ns | ns | ns |
| Ugt2a3 | ns | ns | 7.63E-12 | -2.43 | 1.09E-04 | -1.54 | 1.01E-05 | -1.66 | 0.016 | -1.30 | 5.00E-06 | -1.69 |
| Ugt2b1 | 3.36E-05 | -1.46 | 8.14E-06 | -1.51 | 0.003 | -1.30 | 8.81E-10 | -1.85 | 1.55E-06 | -1.57 | 6.34E-14 | -2.28 |
| Ugt2b34 | ns | ns | ns | ns | 0.037 | -1.15 | 0.004 | -1.22 | 0.005 | -1.21 | 2.36E-05 | -1.35 |
| Ugt2b35 | 0.021 | -1.34 | 0.033 | -1.31 | ns | ns | ns | ns | ns | ns | 6.18E-04 | 1.57 |
| Ugt2b36 | ns | ns | ns | ns | ns | ns | ns | ns | ns | ns | 0.010 | -1.38 |
| Ugt2b37 | ns | ns | 0.011 | -1.50 | 3.84E-05 | -2.00 | 2.04E-05 | -2.06 | 2.09E-04 | -1.85 | 3.12E-04 | -1.82 |
| Ugt2b38 | ns | ns | 4.31E-06 | -1.53 | 0.032 | -1.20 | 7.79E-07 | -1.59 | ns | ns | 9.12E-06 | -1.51 |
| Ugt2b5 | ns | ns | 0.003 | -1.41 | ns | ns | 7.30E-07 | -1.84 | ns | ns | 1.18E-09 | -2.20 |
| Ugt3a1 | 1.12E-05 | -2.08 | 7.62E-06 | -2.12 | 2.03E-06 | -2.24 | 2.54E-13 | -4.19 | 4.35E-13 | -4.10 | 1.07E-28 | -22.82 |
| Ugt3a1 | 1.16E-04 | -1.55 | 5.06E-04 | -1.47 | 0.008 | -1.34 | 9.78E-09 | -2.02 | 7.55E-05 | -1.57 | 1.22E-18 | -3.82 |
| Ugt3a1 | 1.56E-04 | -1.55 | 9.01E-04 | -1.46 | 0.007 | -1.36 | 3.84E-08 | -1.98 | 9.36E-05 | -1.58 | 4.91E-18 | -3.80 |
| Ugt3a2 | 0.001 | -1.47 | 3.15E-05 | -1.68 | 0.040 | -1.27 | 4.18E-06 | -1.79 | 0.0004 | -1.54 | 2.50E-14 | -3.13 |
| Ugt8a | ns | ns | ns | ns | ns | ns | ns | ns | ns | ns | 0.009 | -1.24 |

S2 Table. Micro array analysis of the 15 differentially expressed UGT genes. List of the 15 genes encoding UGT, with a modified expression in 1, 3, 5, 7, 9, and 11-week-old *Npc1^-/-^* mice compared to their control littermates. FC: fold-change, ns: not significant.
